# Microbial Diversity Associated with the Pollen Stores of Captive-Bred Bumble Bee Colonies

**DOI:** 10.3390/insects11040250

**Published:** 2020-04-16

**Authors:** Prarthana S. Dharampal, Luis Diaz-Garcia, Max A. B. Haase, Juan Zalapa, Cameron R. Currie, Chris Todd Hittinger, Shawn A. Steffan

**Affiliations:** 1 Department of Entomology, University of Wisconsin-Madison, Madison, WI 53706, USA; steffan@entomology.wisc.edu; 2 Department of Horticulture, University of Wisconsin-Madison, Madison, WI 53706, USA; diazgarcia@wisc.edu (L.D.-G.); jezalapa@wisc.edu (J.Z.); 3 Instituto Nacional de Investigaciones Forestales, Agricolas y Pecuarias, Aguascalientes 20676, Mexico; 4 Laboratory of Genetics, Genome Center of Wisconsin, DOE Great Lakes Bioenergy Research Center, Wisconsin Energy Institute, J. F. Crow Institute for the Study of Evolution, University of Wisconsin-Madison, Madison, WI 53706, USA; maxabhaase@gmail.com (M.A.B.H.); cthittinger@wisc.edu (C.T.H.); 5 USDA-ARS, Vegetable Crop Research Unit, Madison, WI 53706, USA; 6 Department of Bacteriology, University of Wisconsin-Madison, Madison, WI 53706, USA; Currie@bact.wisc.edu

**Keywords:** microbiome, bee–microbe symbioses, pollen provisions, 16S rRNA gene, ITS gene

## Abstract

The pollen stores of bumble bees host diverse microbiota that influence overall colony fitness. Yet, the taxonomic identity of these symbiotic microbes is relatively unknown. In this descriptive study, we characterized the microbial community of pollen provisions within captive-bred bumble bee hives obtained from two commercial suppliers located in North America. Findings from 16S rRNA and ITS gene-based analyses revealed that pollen provisions from the captive-bred hives shared several microbial taxa that have been previously detected among wild populations. While diverse microbes across phyla Firmicutes, Proteobacteria, Bacteroidetes, Actinobacteria, and Ascomycota were detected in all commercial hives, significant differences were detected at finer-scale taxonomic resolution based on the supplier source. The causative agent of chalkbrood disease in honey bees, *Ascosphaera apis*, was detected in all hives obtained from one supplier source, although none of the hives showed symptoms of infection. The shared core microbiota across both commercial supplier sources consisted of two ubiquitous bee-associated groups, *Lactobacillus* and *Wickerhamiella/Starmerella* clade yeasts that potentially contribute to the beneficial function of the microbiome of bumble bee pollen provisions.

## 1. Introduction

Organisms across the tree of life span a continuum of reliance on microbial symbionts [1,2], collectively referred to as their “microbiome” [3,4]. Host–microbiome interactions can range from mutualistic to pathogenic [2] depending on the taxonomic and functional composition of the microbiome. Non-pathogenic symbionts allow hosts to obtain nutrients from inaccessible substrates [5], utilize novel energy sources [6], and thrive in extreme habitats [7]. Hosts that derive nutritional benefits from their symbionts often demonstrate increased dependence on their microbial partners [8]. In some cases, extreme dependence on nutritional mutualists has led to major evolutionary transitions among several eukaryotic hosts [9]. For instance, it has been speculated that bees diversified from their predatory wasp ancestors by acquiring specialized pollen-digesting gut microbiota [10]. Indeed, the overall fitness of social bees depends on a sustained symbioses with their gut microbiome [3,11,12], which influences several aspects of host physiology, including pollen digestion, carbohydrate utilization, and immune function [13,14,15,16,17,18,19]. 

Microbes that are routinely isolated from healthy replicates are likely to have important functions within host environments, and are collectively referred to as the “core microbiota” [20]. Although the core gut microbiome of corbiculate bees, including honey bees [11,21,22,23,24] and bumble bees [12,13,14,22,25,26,27] share several related bacterial taxa, the manner in which these microbes are transferred through generations varies between bee groups. The inheritance of the gut microbiota is influenced by multiple factors including life history strategies [28], foraging preferences [29], and domestication practices [30]. Honey bee colonies are founded by a large number of workers and a queen, and microbial transmission across generations occurs through trophallaxis and other social interactions between nestmates [11,21,23]. In contrast, bumble bees live in annual colonies, founded by a single overwintered queen [31] who transmits her microbiota to the progeny. Therefore, the microbiome of newly initiated bumble bee colonies reflects that of its queen, and this generational inheritance through a single founding individual imposes a transmission bottleneck on the diversity of the gut microbiota [32]. For captive-bred bumble bees, which have fewer opportunities to acquire microbes during foraging, this may result in loss of overall microbiome diversity and/or of key bacterial strains [33,34]. The taxonomic reorganization of the microbial community can lead to subtle shifts in gut microbiome function, and can contribute to lower pathogen resistance and increased infection rates among captive-bred colonies [35]. Indeed, commercially-reared bumble bee colonies often harbor numerous bee pathogens, many of which have been transmitted to healthy populations through contact with contaminated pollen from infected hives [36,37,38,39]. Such pathogen spillover from commercial to wild colonies has caused dramatic population losses and range contractions among several bumble bee species across North America [40,41,42]. 

While the structure and function of the gut microbiome has been well-investigated [11], relatively little is known about the symbioses between bees and the external community of microbes associated with their pollen stores. Pollen-associated microbes play a central role in the development and nutrition of numerous social [43,44] and solitary bees [45]. Since the complex structure of pollen grains pose a digestive challenge for bees [46], raw pollen is initially mixed with nectar, regurgitated enzymes, and microbes, and fermented to varying degrees before being fed to the larvae. Past research in honey bees demonstrates that an external community of microbial symbionts facilitates this enzymatic process [44,47,48,49,50,51,52], enhancing the digestibility and nutrient content of the raw pollen substrate [50,53]. The resulting nutrient-dense pollen provision, (referred to as beebread in honey bees) consists of pre-digested pollen, nectar, and diverse microbes, and forms the sole source of nutrition for the larvae [22]. Nutrients derived from pollen [54] and pollen-associated microbiota [55] within beebread are assimilated into the biomass of overwintering honey bees and stored until spring. Furthermore, these external symbionts are involved in the production of vital macromolecules [50], long term preservation of stored pollen [56,57,58] and disease prevention [59]. 

Compared to honey bees, which store copious amounts of beebread [58], bumble bees do not overwinter and their colonies do not contain vast pollen reserves [60]. Instead, pollen brought back to the colony is quickly consumed by the residents [61], and unlike beebread, there may be less opportunity for microbial pre-digestion. Nevertheless, most pollen-storing species within the genus *Bombus* age their pollen to some degree before feeding it to their larvae [62,63]. In pollen-storing species, including *B. impatiens* and *B. terrestris,* bee-collected pollen is stored in specialized cells and periodically transferred to the developing larvae inside the brood chambers [63]. The microbe-rich environment of the storage cells suggests that these external symbionts may be involved in some way with larval nutrition. Indeed, empirical estimates reveal that pollen-borne microbes are a significant source of dietary proteins for bumble bees, their contributions often exceeding that of plant-based substrates [55]. Furthermore, direct evidence shows that exposure to fungicides can cause adverse shifts in the microbial community associated with bumble bee pollen provisions and can lead to severe mortality [45,64]. Taken together, growing evidence suggests that the sustained partnership between bumble bees and their external symbionts is integral to colony fitness.

In this study, we explored the bacterial and fungal diversity within the pollen provisions of commercially available bumble bee hives. Commercial hives were purchased from two supplier sources located within different bioregions of North America and reared indoors under semi-sterile conditions. The microbiome of pollen provisions was characterized using Illumina sequencing of the bacterial 16S rRNA and fungal ITS genes. The specific aims of this study were to: (1) characterize and compare the taxonomic diversity of bacteria and fungi within the pollen provisions of bumble bee hives obtained from the two supplier sources, (2) identify the “core microbiome” within the pollen provisions, and (3) use publicly available data to compare the bacterial community of bumble bee pollen provisions with that of the bumble bee gut and other insect–microbe symbioses. 

## 2. Materials and Methods

### 2.1. Rearing and Sample Collection

*Bombus impatiens* hives were purchased from Koppert Biological Systems (Howell, Michigan, USA) in April 2016 (Source K, replicate hives K1, K2, and K3), and Biobest (Leamington, Ontario, Canada) in June 2016 (Source B, replicate hives B1, B2, B3, and B4). Prior to shipment, colonies from both suppliers were raised in captivity at the rearing facility and fed a mixture of commercially obtained pollen and nectar. Once in the laboratory, all hives were acclimatized with trap doors closed within a biosafety cabinet for 7 d. Next, bees were anesthetized by placing them in a −20 °C freezer for 10 min, and the initial number of workers recorded. The mother queen was removed from the hive using sterilized forceps and weighed using a microbalance that was previously sanitized with 90% ethanol. Prior to feeding to the bees, commercially purchased honey bee-collected pollen was sterilized to minimize the chances of introducing any environmentally acquired microbes. Pollen was sterilized by freeze-drying for 72 h, (Labconco, Freezone 2.5+, Kansas City, MO, USA), followed by soaking in 90% ethanol, and drying overnight in a hood under UV light. Sterility was verified by the absence of microbial growth by plating a subsample of the sterilized pollen on general-purpose agar media and incubating at 28 °C for 48 h. Based on previously published values, approximately 4.27 g d^−1^ of sterilized pollen was individually weighed into sterile petri dishes and introduced into the hive through the trap door for 28 d using autoclaved powder funnels [65,66]. At the end of the study, hives were chilled by placing them in a −20 °C freezer for 20 min to record the final worker number and queen weight. Using standard aseptic technique, hives were dissected to exhume pollen provisions from the brood cells. Pollen provisions were stored in PCR collection tubes at −80 °C and transported on ice for DNA analysis.

### 2.2. DNA Isolation and Sequence Processing

One sample weighing approximately 0.3 g of pollen provision was collected from each hive, PCR-amplified, and sequenced at the UW Biotechnology Center. Briefly, DNA from pollen provisions was extracted using lysozyme solution, lysed, and then centrifuged at 10,000 G for 30 s at room temperature (Mo Bio Power Fecal® DNA isolation kit, Hilden, Germany) [45]. The supernatant was transferred to a collection tube and incubated with a protein precipitation solution. After adding an aqueous bind solution, the supernatant was transferred to a spin filter, centrifuged, and eluted in a clean collection tube. The isolated DNA was quantified and normalized to 2 ng μL^−1^ by fluorometric analysis. Reactions were prepared to analyze ITS (fungal), and 16S (bacterial) components of each pollen-provision sample [67]. A nested 2-step PCR protocol was developed using next-generation sequencing libraries targeting the 16S rRNA V3/V4 variable region and ITS/5.8s rRNA spacer region [68,69]. Region specific primers were modified to add sequencer-specific adapter overhang nucleotide sequences to the gene-specific sequences [70,71]. Following amplification, library size was verified and quantified by electrophoretic mobility, and cleaned using solid phase reversible immobilization beads. The 16S and ITS amplicons were pooled quantitatively to create a single amplicon pool for each sample. Sequencer specific adapters and sample specific dual indexes were added using the specific primers and PCR amplified [45]. The quality and quantity of the finished libraries was assessed using electrophoretic mobility, and fluorometry, respectively. All libraries were standardized and pooled prior to sequencing. All 16S rRNA and ITS sequence data were processed in Mothur v.1.38.0 [72]. After merging both PE libraries into single contigs, homopolymers, chimeric sequences, ambiguous sequences, and sequences larger than expected were removed. Unique filtered sequences from the 16S rRNA gene were aligned to the SILVA SSU database Version 123 [73]; the sequences from the ITS gene were aligned to the UNITE database [74]. A distance matrix between successfully aligned sequences was calculated for further OTU assignation using the average neighbor method used in Mothur. Finally, the abundance of each OTU was computed using the make.shared command with label = 0.03 (97% similarity). OTU tables for each gene were imported into R [75] for further statistical analysis (Supplementary Material, Table S1).

### 2.3. Community Analysis

Bacterial, fungal, and overall microbial community structure was visualized using nonmetric multidimensional scaling (NMDS) of the Bray-Curtis dissimilarity coefficient [76]. The generalized and variance-adjusted weighted Unifrac distances between each library were calculated using the R package GUNIFRAC [77], and the PermanovaG function of the same package was used to determine the differences in the fungal and bacterial communities between the two commercial sources. Shannon’s diversity and Pielou’s evenness metrics were computed and compared using the R package Vegan [78]. Microbial OTUs that showed > 50% prevalence and > 1% relative abundance across all replicates within each source were assigned as “core microbiota” [79]. The strength of association between the relative abundance of microbial taxa present in all hives was calculated by Pearson correlation analysis using the corrplot package in R [80]. The bacterial community of bumble bee pollen provisions reported in this study was compared to publicly available data from other insect–microbe symbioses that also amplified the V3/V4 regions of the 16S rRNA gene [34,81,82,83,84]. Clustering of bacterial communities from all selected studies was visualized by NMDS analysis based on the proportions table using the metaMDS function in the R package Vegan.

## 3. Results

**Figure 1:**
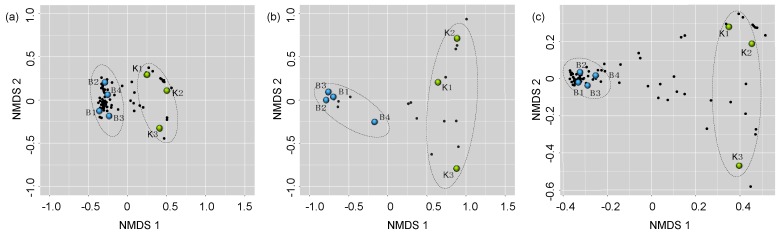
Nonmetric multidimensional scaling of (**a**) bacterial, (**b**) fungal, and (**c**) overall microbial community compositions of pollen provisions from Source K (green circles), and Source B (blue circles). Points that are close together are more similar in diversity and abundance to one another than points that are far apart. Each black circle represents a unique microbial taxon.

Permutation multivariate analysis of variance PermanovaG indicated that the bacterial (*F_1,4_* = 15.81, *p* = 0.035) and fungal (*F_1,4_* = 5.54; *p* = 0.011) community of bumble bee pollen provisions varied significantly between supplier sources. The bacterial community of Source K had significantly lower diversity (H) (*t_5_* = −6.24, *p* = 0.03) and species evenness (J) (*t_5_* = −6.35, *p* < 0.01) compared to Source B. In contrast, the fungal community of Source B had significantly lower diversity (*t_5_* = 2.87, *p* = 0.04) and species evenness (*t_5_* = 2.86, *p* = 0.04) compared to Source K (Supplementary Material, Table S2). NMDS analysis revealed that the bacterial, fungal, and overall microbial communities clustered more closely in replicate hives from the same supplier source, compared to hives from different sources (Figure 1). 

**Figure 2:**
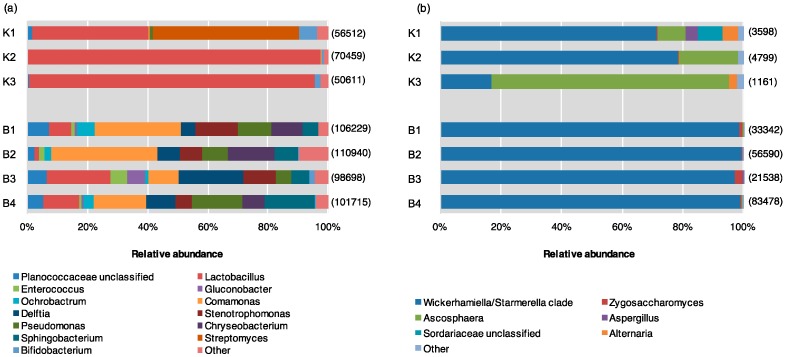
Relative abundance of (**a**) bacterial and (**b**) fungal genera within pollen provisions of indoor–reared bumble bee colonies from Source K (*N* = 3), and Source B (*N* = 4). Sequences belonging to different microbial genera are shown in different colors. The total number of sequences isolated is displayed to the right of each library.

Bacteria detected from pollen provisions included members of four phyla (Firmicutes, Proteobacteria, Bacteroidetes, Actinobacteria). Source K had higher abundance of Firmicutes, mainly belonging to the genus *Lactobacillus*. Actinobacteria included genera *Streptomyces* and *Bifidobacterium,* while there were minimal contributions from Proteobacteria and Bacteroidetes. In contrast, Source B had greater abundance of Proteobacteria (genera *Ochrobactrum* within Alphaproteobacteria, *Comamonas* and *Delftia* within Betaproteobacteria, and *Stenotrophomonas* and *Pseudomonas* within Gammaproteobacteria), and Bacteroidetes (*Chryseobacterium* and *Sphingobacterium*). While there was a lower abundance of *Lactobacillus* compared to Source K, Source B included other Firmicutes, including *Enterococcus* and an unclassified member within the Planococcaceae family (Figure 2a, Supplementary Material, Table S3).

All sequences identified from ITS-based analyses belonged to the phylum Ascomycota, predominantly represented by classes Eurotiomycetes and Saccharomycetes (the only described class in the budding yeast subphylum Saccharomycotina). However, significant differences were noted at the genus rank between supplier sources; Source K had the greatest abundance from Saccharomycetes, dominated by yeasts of the *Wickerhamiella/Starmerella* (W/S) clade (subphylum Saccharomycotina) with minor contributions from *Zygosaccharomyces*. Source K hives also contained substantial amounts of members within genera *Ascosphaera,* including *Ascosphaera apis,* the causative agent of chalkbrood disease in honey bees. Minor contributions were also noted from *Aspergillus, Alternaria,* and an unclassified member of Sordariaceae. More than 99% of the fungal community of Source B hived consisted of members within Saccharomycetes, especially W/S clade yeasts, and *Zygosaccharomyces* at lower abundance. The remainder of the Source B fungal community included trivial contributions (<0.5%) from members within genus *Ascosphaera* and *Aspergillus* (Figure 2b).

**Figure 3:**
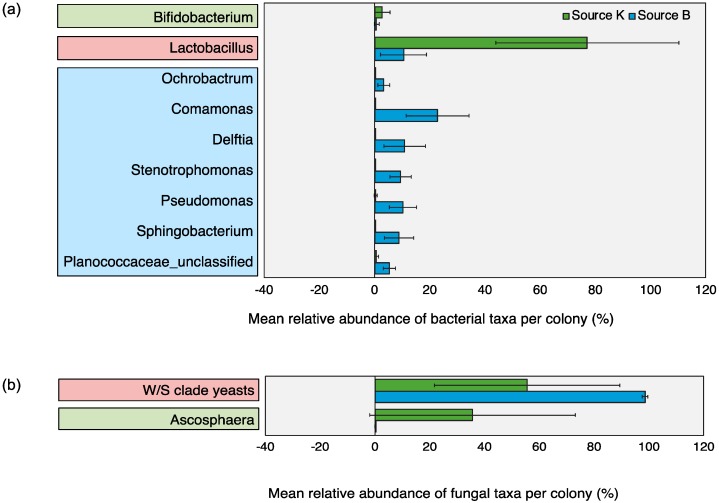
Relative abundance of core (**a**) bacterial and (**b**) fungal taxa detected within pollen provisions of bumble bee hives from Source K (green bars) and Source B (blue bars) (Mean ± 1SD). Microbial OTUs that showed > 50% prevalence and >1% relative abundance across all replicates within each source were assigned as core microbiota (based on Graystock et al [79]). Core microbiota of Source K hives are enclosed within green boxes, that of Source B hives are enclosed within blue boxes, and the shared core microbiota across both sources are enclosed within red boxes.

**Figure 4:**
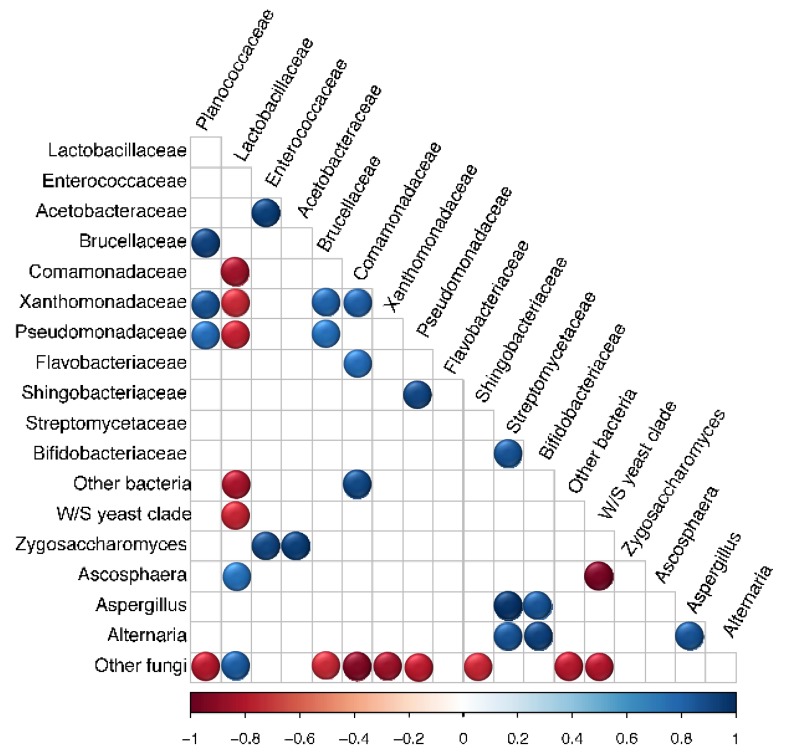
Pearson correlation matrix showing the relative abundance of microbial taxa within bumble bee pollen provisions from both sources (*N* = 7). Significant positive and negative correlations are displayed in blue and red circles, respectively (*p* < 0.05). Color intensity is proportional to the correlation coefficients.

Microbial OTUs that showed > 50% prevalence and > 1% relative abundance across all replicates within each source were assigned as “core microbiota” [79]. This cut off criterion was chosen based on previous work [79] and did not represent an established biological parameter. Based on this characterization, the core microbiota of Source K included two fungal and two bacterial taxa, whereas that of Source B consisted of one fungal and eight bacterial taxa. The shared core microbiota between the two sources consisted of only two groups, *Lactobacillus* and W/S clade yeasts (Figure 3). Pearson correlation analysis revealed significant associations between the relative abundance of multiple pairs of microbial taxa across all hives (Figure 4). Of these, the strongest positive correlations were detected between Enterococcaceae and Acetobacteraceae (*r* = 0.956, *p* < 0.001), Zygosaccharomyces and Acetobacteraceae (*r* = 0.959, *p* < 0.001), and Streptomycetaceae and Aspergillus (*r* = 0.995, *p* < 0.001). The strongest negative correlations were detected between W/S clade yeasts and Ascosphaera (*r* = −0.974, *p* < 0.001), and Lactobacillaceae and Comamonadaceae (*r* = −0.867, *p* < 0.01). 

Hives from both sources showed a significant increase in the number of workers (Source K: *t*_2_ = −8.07, *p* = 0.02; Source B: *t*_2_ = −6.23, *p* < 0.01), although the percent increase was significantly higher in Source B compared to Source K (*t*_5_ = −3.26, *p* = 0.02). However, no statistical difference was noted between the initial and final weights of the mother queen for either source over time (Source K: *t*_2_ = 0.37, *p* = 0.75; Source B: *t*_3_ = −0.43, *p* = 0.69) (Supplementary Material, Table S4).

## 4. Discussion

Our findings revealed that the pollen stores of commercial bumble bee hives harbor several microbial taxa that are frequently associated with diverse bee species and their environment. Unlike the highly conserved bee gut microbiome, the taxonomic composition of bumble bee stored pollen varied significantly based on the commercial supplier sources. Both the bacterial and fungal communities were more similar among replicate hives obtained from the same source, compared to hives from different sources. Hives from both sources shared several members within phyla Firmicutes, Proteobacteria, Bacteroidetes, Actinobacteria, and Ascomycota. However, there were significant differences between the microbial community structure at lower ranks based on the commercial supplier. While Source K supported a significantly more diverse and even fungal community, Source B supported a significantly more diverse and even bacterial community. We speculate that the unique characteristics of each supplier, including the microbiome of the foundress queens and pollen diet used during captive breeding may have contributed to the differences between the microbial communities of bumble bee pollen stores.

Bumble bee colonies are founded by a single overwintered queen, and only the microbes colonizing the queen are expected to be transferred to the residents of the newly initiated colony. The single-queen generational inheritance can cause severe transmission bottlenecks, leading to a loss in microbial diversity and emergent function [30,85,86]. Commercial colonies, which have fewer opportunities to acquire microbes from their foraging environment, may be more vulnerable to such losses in microbial diversity. Indeed, past work has shown that commercial bumble bee hives, harbor a subset of the microbes associated with wild-caught colonies [33,34], and that subtle shifts in their gut microbiome may cause increased disease susceptibility among domesticated lineages [30]. Although there is limited evidence at this time, it is possible that captive-bred colonies experience similar alterations to the microbial community of their pollen stores, and that the loss of key symbiotic taxa could contribute to the increased pathogen loads observed within commercial bumble bee hives.

The microbial OTUs that showed > 50% prevalence and > 1% relative abundance across all replicate hives within each source were assigned as “core” groups. This cut off criterion was chosen based on previous work [79] and did not represent an established biological parameter. Based on this criterion, the shared core microbiota of the two commercial sources was restricted to only two putative pollen fermenters, *Lactobacillus* and yeasts of the *Wickerhamiella/Starmerella* (W/S) clade. Past research indicates that both groups perform unique beneficial functions for their hosts, including fermentation and long-term preservation of hive-stored pollen across managed and wild bee populations [24,44,49,50,57,76,87,88,89]. *Lactobacilli* are ubiquitously associated with multiple genera of bees, spanning a variety of locations, environmental conditions, and genetic traits [23,79,90]. Prior investigations into the gut and pollen microbiome of corbiculate bees indicate that *Lactobacillus* play a central role in bee metabolism, nutrition, and immune function, suggesting a similar functionality within bumble bee pollen provisions as well. Members of the genus *Lactobacillus* within the honey bee gut and hive environment mediate carbohydrate metabolism [11], defend against pathogens [44], produce essential secondary metabolites [43], and prevent spoilage of hive-stored food [56]. Culture-based studies with small number of isolates have previously hinted at the close association of W/S clade yeasts within diverse bee taxa [48,91,92]. Past work in honey bees [47], bumble bees [93], and stingless bees [94] has speculated that fermentative yeasts, including those belonging to the W/S clade [95], play an important role in bee nutrition. Our present study is the first to confirm and extend these findings using culture-independent sequencing techniques.

### 4.1. Bacterial Community

**Figure 5:**
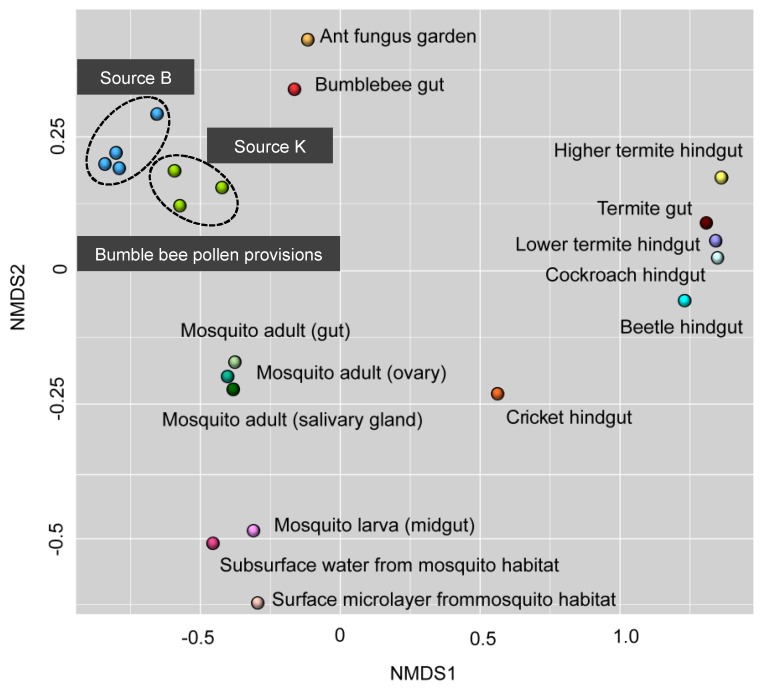
Comparative analysis of nonmetric multidimensional scaling using 16S sequencing data obtained from past studies of insect–microbe symbiosis. Points that are close together are more similar in diversity and abundance to one another than points that are far apart. Each symbol represents the bacterial community detected from a unique insect–microbe symbiosis. See text for references.

Hives from both sources harbored bacterial taxa that were consistent with known bee- and pollen-associated groups (e.g., Streptomycetaceae, Bifidobacteriaceae, Bacillales, Flavobacteriaceae, and Acetobacteraceae) [12,22,24,32]. Many of these groups are specific to beebread, and aid in maintaining general hygiene, inhibit parasites and pathogens, and prevent spoilage of stored pollen [34,52,76]. In addition to *Lactobacillus,* the core bacterial community of Source K included Bifidobacterium, which is speculated to be involved with pollen digestion in honey bees [22]. One library within Source K included a high abundance of *Streptomyces*, which has been previously detected in pollen stores of honey bees [76] and stingless bees [96], and is known to defend against pathogens including *Paenibacillus larvae* and *Melisococcus plutonius* that cause American foulbrood and European foulbrood disease, respectively [97,98]. The bacterial community of Source B was significantly more diverse compared to Source K, and contained higher abundances of Betaproteobacteria, Gammaproteobacteria, and Bacteroides within the Flavobacteriaceae and Sphingobacteriaceae family, which have been previously detected in beebread [43,88]. Source B hives also included Alphaproteobacteria belonging to the Acetobacteraceae family, a osmotolerant and acid resistant bacteria that serves as a source of inoculum for the early instar larval gut of honey bees [99]. The communities of pollen-associated bacteria identified in our study were distinct from those detected from other insect–microbe symbioses. This could suggest a high phylogenetic specificity of the bacterial community within bumble bee pollen provisions (Figure 5). Furthermore, the bacterial community of stored pollen from both commercial sources were more similar to each other than they were to the bumble bee gut microbiome [11,34]. This divergence between the microbiome of bumble bee gut and pollen provision could suggest that these two microhabitats support distinct, and well-adapted communities that likely perform varying roles within their respective environments [22,23,25,43,76]. 

### 4.2. Fungal Community

The core fungal microbiota within pollen provisions from both sources were dominated by W/S clade yeasts. Budding yeasts are ubiquitous within pollen provisions consumed by healthy larvae of social and solitary bees, and appear to be vital for bee nutrition [47,48,91]. All hives also contained other common bee-associated fungi, including representatives from Eurotiomycetes, Sordariomycetes, and Dothideomycetes at lower abundances [45,48,93]. However, there were significant differences in the fungal communities between commercial sources. Hives from Source K showed a high relative abundance of Ascosphaeraceae, including the honey bee pathogen, *Ascosphaera apis*, which causes symptoms similar to that of chalkbrood disease in bumble bees [100] (Supplementary Material, Table S5). Although none of the Source K hives showed signs of chalkbrood, it is interesting to note that they had a significantly slower growth rate compared to Source B hives. Since previous work has detected chalkbrood disease within living, asymptomatic larval honey bees [101,102], it is possible that the decreased colony growth rate of Source K hives was indicative of a subclinical *A. apis* infection. 

In contrast to Source K, the fungal community of Source B hives had a higher abundance of other non-pathogenic fungal taxa, with W/S clade yeasts contributing to over 95% of the overall community. Past research in honey bees indicates that beebread from chalkbrood-resistant colonies have a higher abundance of non-pathogenic yeasts and molds, many of which produce inhibitory antimycotics against *A. apis* [103]. Our data from pairwise comparisons of microbial abundance within bumble bee pollen provisions was consistent with this earlier finding. Across all significant associations identified in this study, the strongest negative correlation was observed between the relative abundance of non-pathogenic W/S clade yeasts and *Ascosphaera sp.* (Figure 4). Although W/S clade yeasts may not directly contribute to increased resistance to chalkbrood disease, it is possible that their interactions with other microbial taxa can indirectly affect the abundance of *A. apis* [103]. Members within the genus *Bacillus, Bifidobacterium*, and *Streptomyces* present within the gut and hive environments of social bees produce antibiotics, antifungals and secondary metabolites, which prevent fungal infections in bees [16,17,24,76,104,105]. Based on these past studies, it is possible that greater diversity in the overall and core bacterial community could explain the lower loads of pathogenic *Ascosphaera* observed within Source B hives.

### 4.3. Implications for Bumble Bee Health

There are several documented cases of pathogen transmission from honey bees to bumble bees in Europe and North America [106,107,108,109,110,111]. The honey bee pathogen *A. apis* has been detected within the colonies of many captive-reared bumble bees species, including *B. griseocollis, B. nevadensis*, *B. vosnesenskii*, and *B. terrestris* [112]. A recent study reveals that infected colonies of *B. terrestris* showed symptoms similar to that of chalkbrood disease in honey bees [100]. However, this study is the first to report the appearance of *A. apis* within the hive-stored pollen of commercially reared *B. impatiens*, which adds to the growing concern about pathogen-driven bumble bee decline [40]. Our study shows that while all hives were reared under similar laboratory conditions using sterilized pollen diets, the abundance of *A. apis* was substantially higher in Source K hives. On average, *A. apis* contributed to nearly 30% of the fungal community in hives obtained from Source K, compared to 0.1% in those from Source B. This suggests that *A. apis* contamination could likely be linked to the supplier of Source K hives, and that these hives may have been exposed to the pathogen at the rearing facility during captive breeding, prior to commercial distribution.

One potential route of infection among commercially reared bumble bees is the use of infected foundress queens. Captive colonies are often founded by wild-caught queen bees that may have been exposed to various pathogens within their natural environment before capture [113,114,115,116]. When such infected queens are used for commercial rearing, there is a high risk of pathogen transmission into the newly initiated colonies [117]. Another potential route of exposure is the use of infected honey bee-collected pollen as diet for the developing colonies reared in captivity [118]. Contaminated honey bee pollen, which often carries bumble bee pathogens, has been reported to cause widespread infections at commercial rearing facilities and can lead to rapid declines in bumble bee populations [112,118,119,120,121]. The risk of pathogen spillover from the release of such infected colonies to native bee populations poses a significant threat to the health of wild bees [35,38,41,118,120,122,123].

Colony initiation in bumble bees occurs anew every year through a single overwintered founding queen, whereas honey bee colonies are formed by large interacting groups of overwintering bees. It is hypothesized that while trophallaxis and other social interactions promote recurrent gut colonization among honey bees [124], the inheritance of microbes through a single founding queen contributes to the high heterogeneity of the bumble bee gut microbiome [32,125]. Each generation of bumble bees can acquire a unique microbiome from the new founding queen, and these colony-level differences can be further amplified during the extreme isolation experienced within artificial rearing facilities. Since foragers of captive-bred colonies have less opportunity to acquire microbes from the environment, the microbial community within the hives used in this study likely differed from that of wild populations. Given that the microbial communities varied significantly between supplier sources, it is possible that our results could have stemmed from the inheritance of unique microbiota specific to the founding queens, and/or microbes within distinct honey bee-collected pollen diets used at the two rearing facilities. Indeed, the commercial suppliers used in this study were located in different countries within North America and may be subject to different policies and regulations pertaining to local land management, agricultural practices, and pesticide regulations within their respective landscapes. Such unique characteristics of each breeding facility based on supplier location could be important in explaining the source-specific differences reported in our study. Another factor that may have influenced our results involves the controlled laboratory conditions of the experiment. The natural variability experienced by wild bees is likely to promote a high diversity of environmentally acquired microbes. In contrast, the hives used in this study were reared indoors under semi-sterile conditions and fed sterilized pollen, leaving minimal opportunity for the influx of environmental- and diet-derived microbes. This could have potentially led to an underestimation of the microbial diversity associated with the pollen provisions of natural bee populations. 

## 5. Conclusions

Our findings indicated that while the microbial community of bumble bee pollen provisions were quite variable across commercial sources, the shared core microbiota across both suppliers was restricted to only two groups: *Lactobacillus* and W/S clade yeasts. Although the exact composition of the “healthy” microbiome remains unclear, the reshuffling of key taxa within the community of these external symbionts can have unpredictable impacts on commercial bee hives. Future investigations comparing pollen stores of captive and wild bumble bee colonies could reveal critical links between microbiome structure and colony fitness, and help establish a predictive framework to conserve bee populations. 

## Supplementary Materials

The following are available online at https://www.mdpi.com/2075-4450/11/4/250/s1. Table S1: Unique OTUs, taxonomy, and sequence read number. Each row is a unique OTU, and each column is a pollen provision library. Table S2: Shannon’s diversity (H’) and Pielou’s evenness (J’) of bacterial and fungal community; Table S3: Distribution of sequencing reads of bacterial and fungal taxa at the family and order rank respectively; Table S4: Colony demography and mother queen weights of hives; Table S5: Top blast hit (Mothur) of genus *Ascosphaera* isolated in this study. All sequences are deposited in GenBank under accession number PRJNA414625.
