# Supplementary material for: Microbial Diversity Associated with the Pollen Stores of Captive-Bred Bumble Bee Colonies
Source: Insects. 2020 Apr 16;11(4):250. doi: 10.3390/insects11040250 (PMC7240610; doi:10.3390/insects11040250)
Supplement: Supplementary file 1 [file insects-11-00250-s001.pdf]

Article submitted to the Special Issue of Insects: Bees and Their Symbionts

Online Resource 2

Supplemental Information

Manuscript title: **Microbial diversity associated with the pollen stores of captive-bred bumble bee colonies**

Prarthana S. Dharampal[1*], Luis Diaz-Garcia[2, 3], Max A. B. Haase[4], Juan Zalapa[2, 5], Cameron R. Currie[6], Chris Todd Hittinger[4], Shawn A. Steffan[1, 5]

1 Department of Entomology, University of Wisconsin-Madison, Madison 53706, USA
2 Department of Horticulture, University of Wisconsin-Madison, Madison 53706, USA
3 Instituto Nacional de Investigaciones Forestales, Agricolas y Pecuarias, Aguascalientes 20676, Mexico.
4 Laboratory of Genetics, Genome Center of Wisconsin, DOE Great Lakes Bioenergy Research Center, Wisconsin Energy Institute, J. F. Crow Institute for the Study of Evolution, University of Wisconsin-Madison, Madison 53706, USA
5 USDA-ARS, Vegetable Crop Research Unit, Madison 53706, USA,
6 Department of Bacteriology, University of Wisconsin-Madison, Madison 53706, USA
* Correspondence: pghosh6@wisc.edu

Table 1. Unique OTUs, taxonomy, and sequence read number. Each row is a unique OTU, and each column is a pollen provision library.

Fungal_OTU_0.03.

Group	Taxonomy	A1	A2	A3	B1	B2	B3	B4
Otu0001	k__Fungi;p__Ascomycota;c__Saccharomycetes;o__Saccharomycetales;f__Saccharomycetales_fam_Incertae_sedis;g__Candida;s__Candida_magnoliae;Candida_magnoliae;	2140	2921	167	23948	41070	17571	68106
Otu0002	k__Fungi;p__Ascomycota;c__Saccharomycetes;o__Saccharomycetales;f__Saccharomycetales_fam_Incertae_sedis;g__Candida;s__Candida_magnoliae;Candida_magnoliae;	13	2	0	2295	1097	647	11569
Otu0003	k__Fungi;p__Ascomycota;c__Saccharomycetes;o__Saccharomycetales;f__Saccharomycetales_fam_Incertae_sedis;g__Candida;s__Candida_magnoliae;Candida_magnoliae;	391	798	27	6399	3012	2468	2500
Otu0004	k__Fungi;p__Ascomycota;c__Saccharomycetes;o__Saccharomycetales;f__Saccharomycetales_fam_Incertae_sedis;g__Candida;g__Candida_unclassified;	0	1	0	18	10460	7	19
Otu0005	k__Fungi;p__Ascomycota;c__Eurotiomycetes;o__Ascosphaerales;f__Ascosphaeraceae;g__Ascosphaera;s__Ascosphaera_apis;Ascosphaera_apis;	291	184	899	12	4	9	293
Otu0006	k__Fungi;p__Ascomycota;c__Saccharomycetes;o__Saccharomycetales;f__Saccharomycetales_fam_Incertae_sedis;g__Candida;s__Candida_magnoliae;Candida_magnoliae;	15	18	0	128	254	78	449
Otu0007	k__Fungi;p__Ascomycota;c__Saccharomycetes;o__Saccharomycetales;f__Saccharomycetaceae;g__Zygosaccharomyces;s__Zygosaccharomyces_rouxii;Zygosaccharomyces_rouxii;	0	1	0	366	192	22	220
Otu0008	k__Fungi;p__Ascomycota;c__Saccharomycetes;o__Saccharomycetales;f__Saccharomycetaceae;g__Zygosaccharomyces;g__Zygosaccharomyces_unclassified;	16	16	2	32	30	572	120
Otu0009	k__Fungi;p__Ascomycota;c__Eurotiomycetes;o__Ascosphaerales;f__Ascosphaeraceae;g__Ascosphaera;g__Ascosphaera_unclassified;	31	745	1	0	0	0	0
Otu0010	k__Fungi;p__Ascomycota;c__Saccharomycetes;o__Saccharomycetales;f__Saccharomycetales_fam_Incertae_sedis;g__Candida;s__Candida_magnoliae;Candida_magnoliae;	0	0	0	71	274	121	7
Otu0011	k__Fungi;p__Ascomycota;c__Sordariomycetes;o__Sordariales;f__Sordariaceae;f__Sordariaceae_unclassified;f__Sordariaceae_unclassified;	281	0	0	0	0	0	0
Otu0012	k__Fungi;p__Ascomycota;c__Dothideomycetes;o__Pleosporales;f__Pleosporaceae;g__Alternaria;g__Alternaria_unclassified;	183	4	29	0	0	0	0
Otu0013	k__Fungi;p__Ascomycota;c__Eurotiomycetes;o__Eurotiales;f__Trichocomaceae;g__Penicillium;g__Penicillium_unclassified;	0	0	0	3	47	5	75
Otu0014	k__Fungi;p__Ascomycota;c__Eurotiomycetes;o__Eurotiales;f__Trichocomaceae;g__Aspergillus;s__Aspergillus_terreus;Aspergillus_terreus;	123	0	0	0	0	0	0
Otu0015	k__Fungi;p__Ascomycota;c__Saccharomycetes;o__Saccharomycetales;f__Saccharomycetales_fam_Incertae_sedis;g__Candida;s__Candida_magnoliae;Candida_magnoliae;	0	0	0	41	42	10	0
Otu0016	k__Fungi;p__Ascomycota;c__Eurotiomycetes;o__Eurotiales;f__Trichocomaceae;g__Aspergillus;g__Aspergillus_unclassified;	0	0	0	2	56	7	0
Otu0017	k__Fungi;p__Ascomycota;c__Saccharomycetes;o__Saccharomycetales;f__Saccharomycetaceae;g__Zygosaccharomyces;s__Zygosaccharomyces_rouxii;Zygosaccharomyces_rouxii;	0	0	0	0	0	7	29
Otu0018	k__Fungi;p__Ascomycota;c__Saccharomycetes;o__Saccharomycetales;f__Saccharomycetales_fam_Incertae_sedis;g__Candida;s__Candida_magnoliae;Candida_magnoliae;	0	0	0	4	8	1	19
Otu0019	k__Fungi;p__Ascomycota;c__Eurotiomycetes;o__Eurotiales;f__Trichocomaceae;g__Aspergillus;g__Aspergillus_unclassified;	28	0	0	0	0	0	0
Otu0020	k__Fungi;p__Ascomycota;p__Ascomycota_unclassified;p__Ascomycota_unclassified;p__Ascomycota_unclassified;p__Ascomycota_unclassified;p__Ascomycota_unclassified;	0	28	0	0	0	0	0
Otu0021	k__Fungi;p__Ascomycota;c__Saccharomycetes;o__Saccharomycetales;f__Saccharomycetales_fam_Incertae_sedis;g__Candida;s__Candida_magnoliae;Candida_magnoliae;	0	0	0	4	7	3	12
Otu0022	k__Fungi;p__Ascomycota;c__Sordariomycetes;o__Sordariales;f__Chaetomiaceae;g__Chaetomium;s__Chaetomium_strumarium;Chaetomium_strumarium;	26	0	0	0	0	0	0
Otu0023	k__Plantae;p__unclassified_Plantae;c__unclassified_Plantae;o__unclassified_Plantae;f__unclassified_Plantae;g__unclassified_Plantae;s__Plantae_sp;Plantae;	7	4	11	0	0	0	0
Otu0024	k__Fungi;p__Ascomycota;c__Saccharomycetes;o__Saccharomycetales;f__Saccharomycetales_fam_Incertae_sedis;g__Candida;s__Candida_sorbosivorans;Candida_sorbosivorans;	9	12	0	0	0	0	0
Otu0025	k__Fungi;p__Ascomycota;c__Saccharomycetes;o__Saccharomycetales;f__Saccharomycetales_fam_Incertae_sedis;g__Kodamaea;s__Kodamaea_ohmeri;Kodamaea_ohmeri;	13	8	0	0	0	0	0
Otu0026	k__Fungi;p__Ascomycota;c__Saccharomycetes;o__Saccharomycetales;f__Saccharomycetales_fam_Incertae_sedis;g__Candida;s__Candida_magnoliae;Candida_magnoliae;	1	0	0	2	12	0	3
Otu0027	k__Fungi;p__Ascomycota;c__Saccharomycetes;o__Saccharomycetales;f__Saccharomycetales_fam_Incertae_sedis;g__Candida;s__Candida_magnoliae;Candida_magnoliae;	2	14	0	0	1	0	0
Otu0028	k__Fungi;p__Ascomycota;c__Eurotiomycetes;o__Eurotiales;f__Trichocomaceae;g__Aspergillus;g__Aspergillus_unclassified;	2	0	0	0	0	0	14
Otu0029	k__Fungi;p__Ascomycota;c__Saccharomycetes;o__Saccharomycetales;f__Saccharomycetales_fam_Incertae_sedis;g__Candida;s__Candida_magnoliae;Candida_magnoliae;	0	0	0	2	0	0	13
Otu0030	k__Fungi;p__Ascomycota;c__Saccharomycetes;o__Saccharomycetales;f__Saccharomycetales_fam_Incertae_sedis;g__Candida;s__Candida_magnoliae;Candida_magnoliae;	0	0	0	0	7	0	6
Otu0031	k__Plantae;p__unclassified_Plantae;c__unclassified_Plantae;o__unclassified_Plantae;f__unclassified_Plantae;g__unclassified_Plantae;s__Plantae_sp;Plantae;	0	10	3	0	0	0	0
Otu0032	k__Fungi;p__Ascomycota;c__Eurotiomycetes;o__Ascosphaerales;f__Ascosphaeraceae;g__Ascosphaera;s__Ascosphaera_apis;Ascosphaera_apis;	0	0	12	0	0	0	0
Otu0033	k__Fungi;p__Ascomycota;c__Saccharomycetes;o__Saccharomycetales;f__Saccharomycetales_fam_Incertae_sedis;g__Candida;s__Candida_magnoliae;Candida_magnoliae;	0	0	0	2	1	0	8
Otu0034	k__Fungi;k__Fungi_unclassified;k__Fungi_unclassified;k__Fungi_unclassified;k__Fungi_unclassified;k__Fungi_unclassified;k__Fungi_unclassified;	0	7	3	0	0	0	0
Otu0035	k__Plantae;p__unclassified_Plantae;c__unclassified_Plantae;o__unclassified_Plantae;f__unclassified_Plantae;g__unclassified_Plantae;s__Plantae_sp;Plantae;	9	0	0	0	0	0	1
Otu0036	k__Fungi;p__Ascomycota;c__Saccharomycetes;o__Saccharomycetales;f__Saccharomycetales_fam_Incertae_sedis;g__Candida;s__Candida_magnoliae;Candida_magnoliae;	0	0	0	2	0	2	4
Otu0037	k__Fungi;p__Ascomycota;c__Sordariomycetes;o__Sordariales;f__Chaetomiaceae;g__Chaetomium;g__Chaetomium_unclassified;	4	0	0	0	0	0	4
Otu0038	k__Fungi;p__Ascomycota;c__Sordariomycetes;o__Sordariales;f__Sordariaceae;f__Sordariaceae_unclassified;f__Sordariaceae_unclassified;	8	0	0	0	0	0	0
Otu0039	k__Fungi;p__Ascomycota;c__Sordariomycetes;o__Hypocreales;f__Nectriaceae;f__Nectriaceae_unclassified;f__Nectriaceae_unclassified;	6	0	0	0	0	0	1
Otu0040	k__Plantae;p__unclassified_Plantae;c__unclassified_Plantae;o__unclassified_Plantae;f__unclassified_Plantae;g__unclassified_Plantae;s__Plantae_sp;Plantae;	6	0	1	0	0	0	0
Otu0041	k__Fungi;p__Ascomycota;c__Saccharomycetes;o__Saccharomycetales;o__Saccharomycetales_unclassified;o__Saccharomycetales_unclassified;o__Saccharomycetales_unclassified;	0	0	0	3	3	0	0
Otu0042	k__Fungi;p__Ascomycota;c__Saccharomycetes;o__Saccharomycetales;f__Saccharomycetales_fam_Incertae_sedis;g__Candida;s__Candida_magnoliae;Candida_magnoliae;	0	0	0	0	1	5	0
Otu0043	k__Fungi;p__Ascomycota;c__Dothideomycetes;o__Capnodiales;f__Davidiellaceae;g__Cladosporium;g__Cladosporium_unclassified;	1	0	3	0	0	0	1
Otu0044	k__Fungi;p__Ascomycota;c__Saccharomycetes;o__Saccharomycetales;f__Metschnikowiaceae;g__unclassified_Metschnikowiaceae;s__Metschnikowiaceae_sp;Metschnikowiaceae;	0	5	0	0	0	0	0
Otu0045	k__Plantae;p__unclassified_Plantae;c__unclassified_Plantae;o__unclassified_Plantae;f__unclassified_Plantae;g__unclassified_Plantae;s__Plantae_sp;Plantae;	0	2	3	0	0	0	0
Otu0046	k__Fungi;p__Ascomycota;c__Saccharomycetes;o__Saccharomycetales;f__Saccharomycetales_fam_Incertae_sedis;g__Candida;s__Candida_apicola;Candida_apicola;	0	5	0	0	0	0	0
Otu0047	k__Fungi;p__Ascomycota;c__Saccharomycetes;o__Saccharomycetales;f__Saccharomycetales_fam_Incertae_sedis;f__Saccharomycetales_fam_Incertae_sedis_unclassified;f__Saccharomycetales_fam_Incertae_sedis_unclassified;	0	5	0	0	0	0	0
Otu0048	k__Fungi;p__Ascomycota;c__Saccharomycetes;o__Saccharomycetales;f__Saccharomycetales_fam_Incertae_sedis;g__Candida;s__Candida_magnoliae;Candida_magnoliae;	0	0	0	5	0	0	0
Otu0049	k__Fungi;k__Fungi_unclassified;k__Fungi_unclassified;k__Fungi_unclassified;k__Fungi_unclassified;k__Fungi_unclassified;k__Fungi_unclassified;	2	0	2	0	0	0	0
Otu0050	k__Fungi;p__Ascomycota;c__Saccharomycetes;o__Saccharomycetales;f__Saccharomycetales_fam_Incertae_sedis;g__Candida;s__Candida_magnoliae;Candida_magnoliae;	0	0	0	1	3	0	0
Otu0051	k__Fungi;p__Ascomycota;c__Saccharomycetes;o__Saccharomycetales;o__Saccharomycetales_unclassified;o__Saccharomycetales_unclassified;o__Saccharomycetales_unclassified;	0	0	0	0	2	2	0
Otu0052	k__Fungi;k__Fungi_unclassified;k__Fungi_unclassified;k__Fungi_unclassified;k__Fungi_unclassified;k__Fungi_unclassified;k__Fungi_unclassified;	0	2	1	0	0	0	1
Otu0053	unknown;unknown_unclassified;unknown_unclassified;unknown_unclassified;unknown_unclassified;unknown_unclassified;unknown_unclassified;	2	2	0	0	0	0	0
Otu0054	k__Fungi;k__Fungi_unclassified;k__Fungi_unclassified;k__Fungi_unclassified;k__Fungi_unclassified;k__Fungi_unclassified;k__Fungi_unclassified;	0	0	4	0	0	0	0
Otu0055	k__Fungi;p__Ascomycota;c__Dothideomycetes;o__Capnodiales;f__Capnodiaceae;g__Capnodium;s__Capnodium_sp;Capnodium;	0	3	0	0	0	0	0
Otu0056	k__Fungi;p__Ascomycota;c__Sordariomycetes;o__Hypocreales;f__Nectriaceae;g__Fusarium;s__Fusarium_brachygibbosum;Fusarium_brachygibbosum;	2	0	1	0	0	0	0
Otu0057	k__Fungi;p__Basidiomycota;c__Tremellomycetes;o__Tremellales;f__Tremellales_fam_Incertae_sedis;g__Dioszegia;s__Dioszegia_sp;Dioszegia;	3	0	0	0	0	0	0
Otu0058	k__Fungi;p__Basidiomycota;c__Tremellomycetes;o__Tremellomycetes_ord_Incertae_sedis;f__Tremellomycetes_fam_Incertae_sedis;g__Moniliella;g__Moniliella_unclassified;	0	0	3	0	0	0	0
Otu0059	k__Fungi;k__Fungi_unclassified;k__Fungi_unclassified;k__Fungi_unclassified;k__Fungi_unclassified;k__Fungi_unclassified;k__Fungi_unclassified;	0	1	0	0	0	0	1
Otu0060	k__Fungi;k__Fungi_unclassified;k__Fungi_unclassified;k__Fungi_unclassified;k__Fungi_unclassified;k__Fungi_unclassified;k__Fungi_unclassified;	0	2	0	0	0	0	0
Otu0061	k__Fungi;k__Fungi_unclassified;k__Fungi_unclassified;k__Fungi_unclassified;k__Fungi_unclassified;k__Fungi_unclassified;k__Fungi_unclassified;	0	2	0	0	0	0	0
Otu0062	k__Fungi;k__Fungi_unclassified;k__Fungi_unclassified;k__Fungi_unclassified;k__Fungi_unclassified;k__Fungi_unclassified;k__Fungi_unclassified;	0	1	0	0	0	0	1
Otu0063	k__Fungi;p__Ascomycota;c__Saccharomycetes;o__Saccharomycetales;f__Saccharomycetales_fam_Incertae_sedis;g__Candida;s__Candida_magnoliae;Candida_magnoliae;	0	0	0	0	2	0	0
Otu0064	k__Fungi;k__Fungi_unclassified;k__Fungi_unclassified;k__Fungi_unclassified;k__Fungi_unclassified;k__Fungi_unclassified;k__Fungi_unclassified;	1	0	1	0	0	0	0
Otu0065	k__Fungi;p__Basidiomycota;c__Tremellomycetes;o__Tremellales;f__Tremellales_fam_Incertae_sedis;g__Hannaella;s__Hannaella_luteola;Hannaella_luteola;	0	0	2	0	0	0	0
Otu0066	k__Fungi;k__Fungi_unclassified;k__Fungi_unclassified;k__Fungi_unclassified;k__Fungi_unclassified;k__Fungi_unclassified;k__Fungi_unclassified;	0	0	2	0	0	0	0
Otu0067	k__Fungi;k__Fungi_unclassified;k__Fungi_unclassified;k__Fungi_unclassified;k__Fungi_unclassified;k__Fungi_unclassified;k__Fungi_unclassified;	1	1	0	0	0	0	0
Otu0068	k__Fungi;p__Ascomycota;c__Eurotiomycetes;o__Ascosphaerales;f__Ascosphaeraceae;g__Ascosphaera;g__Ascosphaera_unclassified;	0	2	0	0	0	0	0
Otu0069	k__Fungi;p__Ascomycota;c__Dothideomycetes;o__Pleosporales;f__Didymellaceae;g__Allophoma;g__Allophoma_unclassified;	1	1	0	0	0	0	0
Otu0070	k__Fungi;p__Ascomycota;c__Sordariomycetes;o__Trichosphaeriales;o__Trichosphaeriales_unclassified;o__Trichosphaeriales_unclassified;o__Trichosphaeriales_unclassified;	2	0	0	0	0	0	0
Otu0071	k__Fungi;p__Ascomycota;c__Saccharomycetes;o__Saccharomycetales;o__Saccharomycetales_unclassified;o__Saccharomycetales_unclassified;o__Saccharomycetales_unclassified;	2	0	0	0	0	0	0
Otu0072	k__Fungi;p__Ascomycota;c__Dothideomycetes;o__Dothideales;f__Dothioraceae;f__Dothioraceae_unclassified;f__Dothioraceae_unclassified;	0	2	0	0	0	0	0
Otu0073	k__Fungi;p__Ascomycota;c__Sordariomycetes;o__Xylariales;f__Amphisphaeriaceae;f__Amphisphaeriaceae_unclassified;f__Amphisphaeriaceae_unclassified;	0	2	0	0	0	0	0
Otu0074	k__Fungi;k__Fungi_unclassified;k__Fungi_unclassified;k__Fungi_unclassified;k__Fungi_unclassified;k__Fungi_unclassified;k__Fungi_unclassified;	0	2	0	0	0	0	0
Otu0075	k__Fungi;k__Fungi_unclassified;k__Fungi_unclassified;k__Fungi_unclassified;k__Fungi_unclassified;k__Fungi_unclassified;k__Fungi_unclassified;	0	2	0	0	0	0	0
Otu0076	k__Fungi;k__Fungi_unclassified;k__Fungi_unclassified;k__Fungi_unclassified;k__Fungi_unclassified;k__Fungi_unclassified;k__Fungi_unclassified;	0	0	2	0	0	0	0
Otu0077	k__Fungi;p__Ascomycota;c__Saccharomycetes;o__Saccharomycetales;f__Saccharomycetales_fam_Incertae_sedis;g__Candida;s__Candida_magnoliae;Candida_magnoliae;	0	0	0	2	0	0	0
Otu0078	k__Fungi;p__Ascomycota;c__Saccharomycetes;o__Saccharomycetales;f__Saccharomycetales_fam_Incertae_sedis;g__Candida;s__Candida_magnoliae;Candida_magnoliae;	0	0	0	0	2	0	0
Otu0079	k__Fungi;p__Ascomycota;c__Saccharomycetes;o__Saccharomycetales;f__Saccharomycetales_fam_Incertae_sedis;g__Candida;s__Candida_magnoliae;Candida_magnoliae;	0	0	0	0	0	0	2
Otu0080	k__Fungi;p__Ascomycota;c__Eurotiomycetes;o__Ascosphaerales;f__Ascosphaeraceae;g__Ascosphaera;g__Ascosphaera_unclassified;	0	2	0	0	0	0	0
Otu0081	k__Fungi;p__Ascomycota;c__Saccharomycetes;o__Saccharomycetales;f__Saccharomycetales_fam_Incertae_sedis;g__Candida;g__Candida_unclassified;	0	0	0	0	2	0	0
Otu0082	k__Fungi;p__Ascomycota;c__Saccharomycetes;o__Saccharomycetales;f__Saccharomycetales_fam_Incertae_sedis;g__Candida;s__Candida_magnoliae;Candida_magnoliae;	0	0	0	0	0	1	1
Otu0083	k__Fungi;p__Ascomycota;c__Saccharomycetes;o__Saccharomycetales;f__Saccharomycetaceae;g__Zygosaccharomyces;g__Zygosaccharomyces_unclassified;	0	0	0	0	1	0	0

Bactiral_OTU_0.03.

Group	Taxonomy	A1	A2	A3	B1	B2	B3	B4
Otu0001	Bacteria;Firmicutes;Bacilli;Lactobacillales;Lactobacillaceae;Lactobacillus;	21881	68559	48081	5159	1071	13146	10628
Otu0002	Bacteria;Proteobacteria;Betaproteobacteria;Burkholderiales;Comamonadaceae;Comamonas;	35	23	8	30361	39286	9631	17836
Otu0003	Bacteria;Proteobacteria;Betaproteobacteria;Burkholderiales;Comamonadaceae;Delftia;	4	8	9	5018	8345	21523	9810
Otu0004	Bacteria;Bacteroidetes;Flavobacteriia;Flavobacteriales;Flavobacteriaceae;Chryseobacterium;	2	0	0	11294	17246	210	7447
Otu0005	Bacteria;Proteobacteria;Gammaproteobacteria;Xanthomonadales;Xanthomonadaceae;Stenotrophomonas;	16	10	0	8751	8050	10314	5248
Otu0006	Bacteria;Cyanobacteria;Chloroplast;Chloroplast_or;Chloroplast_fa;Chloroplast_ge;	11090	7652	12975	40	20	46	100
Otu0007	Bacteria;Bacteroidetes;Sphingobacteriia;Sphingobacteriales;Sphingobacteriaceae;Sphingobacterium;	6	7	0	14	8620	5810	16834
Otu0008	Bacteria;Actinobacteria;Actinobacteria;Streptomycetales;Streptomycetaceae;Streptomyces;	27545	2	7	48	3	0	0
Otu0009	Bacteria;Proteobacteria;Gammaproteobacteria;Pseudomonadales;Pseudomonadaceae;Pseudomonas;	32	0	0	3742	8384	4774	7819
Otu0010	Bacteria;Proteobacteria;Gammaproteobacteria;Pseudomonadales;Pseudomonadaceae;Pseudomonas;	13	3	33	7756	1110	137	8499
Otu0011	Bacteria;Firmicutes;Bacilli;Bacillales;Planococcaceae;	44	12	2	6940	1788	3365	4577
Otu0012	Bacteria;Proteobacteria;Alphaproteobacteria;Rhizobiales;Brucellaceae;Ochrobactrum;	3	0	0	6666	2353	1331	3551
Otu0013	Bacteria;Firmicutes;Bacilli;Lactobacillales;Enterococcaceae;Enterococcus;	0	0	0	1209	1784	5259	662
Otu0014	Bacteria;Actinobacteria;Actinobacteria;Bifidobacteriales;Bifidobacteriaceae;Bifidobacterium;	3373	498	880	232	315	2024	632
Otu0015	Bacteria;Proteobacteria;Alphaproteobacteria;Rhodospirillales;Acetobacteraceae;Gluconobacter;	23	4	5	748	171	5980	846
Otu0016	Bacteria;Firmicutes;Bacilli;Lactobacillales;Lactobacillaceae;Lactobacillus;	39	80	50	1921	565	4541	13
Otu0017	Bacteria;Proteobacteria;Gammaproteobacteria;Xanthomonadales;Xanthomonadaceae;Stenotrophomonas;	49	0	0	6366	65	198	301
Otu0018	Bacteria;Bacteroidetes;Sphingobacteriia;Sphingobacteriales;Sphingobacteriaceae;Sphingobacterium;	0	0	0	5323	52	0	87
Otu0019	Bacteria;Firmicutes;Bacilli;Lactobacillales;Lactobacillaceae;Lactobacillus;	6	0	0	456	126	3213	1487
Otu0020	Bacteria;Actinobacteria;Actinobacteria;Bifidobacteriales;Bifidobacteriaceae;Bombiscardovia;	1267	918	1104	152	151	635	439
Otu0021	Bacteria;Proteobacteria;Alphaproteobacteria;Caulobacterales;Caulobacteraceae;Brevundimonas;	3	0	0	24	1780	55	1024
Otu0022	Bacteria;Firmicutes;Clostridia;Clostridiales;Lachnospiraceae;Lachnoclostridium_5;	0	0	0	270	295	1201	242
Otu0023	Bacteria;Bacteroidetes;Sphingobacteriia;Sphingobacteriales;Sphingobacteriaceae;Nubsella;	0	0	0	4	1510	16	198
Otu0024	Bacteria;Proteobacteria;Betaproteobacteria;Burkholderiales;Alcaligenaceae;	0	0	0	0	126	1459	78
Otu0025	Bacteria;Actinobacteria;Actinobacteria;Micrococcales;Microbacteriaceae;Leucobacter;	5	0	2	208	1215	23	76
Otu0026	Bacteria;Proteobacteria;Gammaproteobacteria;Pseudomonadales;Pseudomonadaceae;Pseudomonas;	537	5	17	0	0	0	704
Otu0027	Bacteria;Firmicutes;Clostridia;Clostridiales;Clostridiaceae_1;Clostridium_sensu_stricto_3;	0	0	0	121	348	452	315
Otu0028	Bacteria;Firmicutes;Bacilli;Bacillales;Bacillaceae;Oceanobacillus;	0	0	0	563	658	15	0
Otu0029	Bacteria;Firmicutes;Bacilli;Bacillales;Paenibacillaceae;Paenibacillus;	0	0	0	191	331	284	268
Otu0030	Bacteria;Proteobacteria;Betaproteobacteria;Burkholderiales;Comamonadaceae;Pseudorhodoferax;	0	0	0	0	573	10	351
Otu0031	Bacteria;Firmicutes;Bacilli;Lactobacillales;Lactobacillaceae;	0	0	0	1	12	827	0
Otu0032	Bacteria;Firmicutes;Clostridia;Clostridiales;Clostridiaceae_1;Clostridium_sensu_stricto_1;	3	0	0	120	199	268	241
Otu0033	Bacteria;Actinobacteria;Actinobacteria;Micrococcales;Microbacteriaceae;Microbacterium;	0	0	0	220	354	25	74
Otu0034	Bacteria;Bacteroidetes;Flavobacteriia;Flavobacteriales;Flavobacteriaceae;Flavobacterium;	0	0	0	2	661	2	0
Otu0035	Bacteria;Firmicutes;Bacilli;Bacillales;Paenibacillaceae;Cohnella;	0	0	0	209	357	0	87
Otu0036	Bacteria;Proteobacteria;Alphaproteobacteria;Sphingomonadales;Sphingomonadales_unclassified;	0	2	0	286	17	16	262
Otu0037	Bacteria;Proteobacteria;Gammaproteobacteria;Enterobacteriales;Enterobacteriaceae;	92	0	0	127	16	144	193
Otu0038	Bacteria;Actinobacteria;Actinobacteria;Corynebacteriales;Nocardiaceae;Rhodococcus;	0	0	0	2	536	10	0
Otu0039	Bacteria;Firmicutes;Bacilli;Bacillales;Paenibacillaceae;Paenibacillus;	0	0	0	293	48	141	18
Otu0040	Bacteria;Proteobacteria;Gammaproteobacteria;Pseudomonadales;Pseudomonadaceae;	447	9	12	0	0	0	0
Otu0041	Bacteria;Firmicutes;Negativicutes;Selenomonadales;Veillonellaceae;Pelosinus;	0	0	0	0	2	454	0
Otu0042	Bacteria;Firmicutes;Bacilli;Lactobacillales;Lactobacillaceae;	0	0	0	0	77	355	2
Otu0043	Bacteria;Proteobacteria;Gammaproteobacteria;Orbales;Orbaceae;	147	56	175	4	0	19	18
Otu0044	Bacteria;Proteobacteria;Gammaproteobacteria;Pseudomonadales;Moraxellaceae;Acinetobacter;	0	15	0	0	352	2	0
Otu0045	Bacteria;Firmicutes;Bacilli;Bacillales;Bacillaceae;Bacillus;	22	0	0	205	1	98	0
Otu0046	Bacteria;Firmicutes;Clostridia;Clostridiales;Lachnospiraceae;	0	0	0	20	148	88	57
Otu0047	Bacteria;Proteobacteria;Alphaproteobacteria;Rhodobacterales;Rhodobacteraceae;Paracocccus;	304	7	0	0	0	0	0
Otu0048	Bacteria;Proteobacteria;Alphaproteobacteria;Sphingomonadales;Sphingomonadaceae;Sphingobium;	0	0	0	0	125	2	140
Otu0049	Bacteria;Firmicutes;Bacilli;Bacillales;Paenibacillaceae;	0	0	0	254	2	0	0
Otu0050	Bacteria;Firmicutes;Negativicutes;Selenomonadales;Veillonellaceae;Pelosinus;	0	0	0	61	94	0	93
Otu0051	Bacteria;Proteobacteria;Gammaproteobacteria;Enterobacteriales;Enterobacteriaceae;Pantoea;	58	0	0	95	13	29	49
Otu0052	Bacteria;Firmicutes;Bacilli;Bacillales;Paenibacillaceae;Brevibacillus;	0	0	0	153	36	36	18
Otu0053	Bacteria;Firmicutes;Bacilli;Bacillales;Paenibacillaceae;Paenibacillus;	0	0	0	37	104	21	52
Otu0054	Bacteria;Firmicutes;Clostridia;Clostridiales;Clostridiaceae_1;Clostridium_sensu_stricto_13;	0	0	0	20	49	113	28
Otu0055	Bacteria;Firmicutes;Erysipelotrichia;Erysipelotrichales;Erysipelotrichaceae;Erysipelatoclostridium;	0	0	0	0	201	4	0
Otu0056	Bacteria;Bacteroidetes;Sphingobacteriia;Sphingobacteriales;Sphingobacteriaceae;Pedobacter;	0	0	0	0	180	0	4
Otu0057	Bacteria;Proteobacteria;Gammaproteobacteria;Pseudomonadales;Moraxellaceae;Acinetobacter;	0	0	0	83	83	2	5
Otu0058	Bacteria;Firmicutes;Clostridia;Clostridiales;Lachnospiraceae;	0	0	0	34	88	28	22
Otu0059	Bacteria;Firmicutes;Bacilli;Bacillales;Paenibacillaceae;Paenibacillus;	0	0	1	140	7	2	0
Otu0060	Bacteria;Proteobacteria;Gammaproteobacteria;Pseudomonadales;Moraxellaceae;Acinetobacter;	32	63	54	0	0	0	0
Otu0061	Bacteria;Proteobacteria;Betaproteobacteria;Neisseriales;Neisseriaceae;Snodgrassella;	24	8	32	3	4	72	5
Otu0062	Bacteria;Firmicutes;Bacilli;Bacillales;Paenibacillaceae;Paenibacillus;	0	0	0	4	137	0	0
Otu0063	Bacteria;Proteobacteria;Alphaproteobacteria;Rhizobiales;Bradyrhizobiaceae;Bosea;	0	0	0	0	18	15	93
Otu0064	Bacteria;Proteobacteria;Alphaproteobacteria;Rhizobiales;Methylobacteriaceae;Methylobacterium;	67	0	0	0	2	0	39
Otu0065	Bacteria;Proteobacteria;Gammaproteobacteria;Gammaproteobacteria_unclassified;Gammaproteobacteria_unclassified;	0	0	0	107	0	0	0
Otu0066	Bacteria;Actinobacteria;Actinobacteria;Pseudonocardiales;Pseudonocardiaceae;Saccharopolyspora;	99	0	0	0	0	0	0
Otu0067	Bacteria;Proteobacteria;Alphaproteobacteria;Rhizobiales;Rhizobiaceae;	0	0	6	0	54	0	39
Otu0068	Bacteria;Cyanobacteria;Chloroplast;Chloroplast_or;Chloroplast_fa;Chloroplast_ge;	51	15	25	0	0	0	0
Otu0069	Bacteria;Firmicutes;Bacilli;Bacillales;Paenibacillaceae;Paenibacillus;	3	0	0	34	8	28	18
Otu0070	Bacteria;Firmicutes;Bacilli;Lactobacillales;Lactobacillaceae;Lactobacillus;	0	0	0	0	0	90	0
Otu0071	Bacteria;Firmicutes;Clostridia;Clostridiales;Ruminococcaceae;Ruminiclostridium_5;	0	0	0	0	41	34	14
Otu0072	Bacteria;Proteobacteria;Alphaproteobacteria;Rhizobiales;	0	0	0	0	33	0	45
Otu0073	Bacteria;Proteobacteria;Gammaproteobacteria;Enterobacteriales;Enterobacteriaceae;	68	0	0	0	0	0	2

Otu0074	Bacteria;Actinobacteria;Actinobacteria;Micrococcales;Micrococcales_unclassified;	3	0	0	0	62	0	0
Otu0075	Bacteria;Firmicutes;Bacilli;Bacillales;Bacillaceae;	0	0	0	10	53	0	0
Otu0076	Bacteria;Actinobacteria;Actinobacteria;Micrococcales;Microbacteriaceae;Agromyces;	0	0	0	0	60	0	0
Otu0077	Bacteria;Cyanobacteria;Chloroplast;Chloroplast_or;Chloroplast_fa;Chloroplast_ge;	14	4	42	0	0	0	0
Otu0078	Bacteria;Proteobacteria;Gammaproteobacteria;Orbales;Orbaceae;Gilliamella;	0	2	0	6	0	26	19
Otu0079	Bacteria;Actinobacteria;Actinobacteria;Micrococcales;Microbacteriaceae;Agromyces;	0	0	0	0	19	5	27
Otu0080	Bacteria;Firmicutes;Bacilli;Bacillales;Paenibacillaceae;Paenibacillus;	0	0	0	8	27	2	9
Otu0081	Bacteria;Firmicutes;Clostridia;Clostridiales;Peptostreptococcaceae;Peptoclostridium;	0	0	0	2	12	31	0
Otu0082	Bacteria;Firmicutes;Bacilli;Bacillales;Paenibacillaceae;Paenibacillus;	0	0	0	22	0	20	0
Otu0083	Bacteria;Proteobacteria;Alphaproteobacteria;Rhizobiales;Hyphomicrobiaceae;Devosia;	0	0	0	0	36	0	0
Otu0084	Bacteria;Firmicutes;Clostridia;Clostridiales;Clostridiaceae_2;Alkaliphilus;	0	0	0	13	0	22	0
Otu0085	Bacteria;Proteobacteria;Alphaproteobacteria;Caulobacterales;Caulobacteraceae;Phenylobacterium;	0	5	0	0	28	0	0
Otu0086	Bacteria;Firmicutes;Clostridia;Clostridiales;Family_XI;Family_XI_ge;	0	0	0	15	7	6	0
Otu0087	Bacteria;Bacteroidetes;Flavobacteriia;Flavobacteriales;Flavobacteriaceae;Flavobacterium;	0	0	0	0	27	0	0
Otu0088	Bacteria;Firmicutes;Clostridia;Clostridiales;Lachnospiraceae;	0	0	0	0	0	25	0
Otu0089	Bacteria;Firmicutes;Bacilli;Bacillales;Paenibacillaceae;Paenibacillus;	0	0	25	0	0	0	0
Otu0090	Bacteria;Firmicutes;Bacilli;Lactobacillales;Lactobacillaceae;Lactobacillus;	0	0	0	0	21	0	0
Otu0091	Bacteria;Proteobacteria;Alphaproteobacteria;Rhizobiales;Hyphomicrobiaceae;Devosia;	5	0	0	0	0	0	16
Otu0092	Bacteria;Firmicutes;Bacilli;Bacillales;Bacillaceae;Bacillus;	14	5	0	0	2	0	0
Otu0093	Bacteria;Bacteroidetes;Flavobacteriia;Flavobacteriales;Flavobacteriaceae;Chryseobacterium;	0	15	6	0	0	0	0
Otu0094	Bacteria;Firmicutes;Bacilli;Bacillales;Paenibacillaceae;Paenibacillus;	0	0	0	9	12	0	0
Otu0095	Bacteria;Firmicutes;Bacilli;Bacillales;Bacillales_unclassified;	15	0	5	0	0	0	0
Otu0096	Bacteria;Firmicutes;Clostridia;Clostridiales;Lachnospiraceae;	0	0	0	0	19	0	0
Otu0097	Bacteria;Firmicutes;Bacilli;Bacillales;Paenibacillaceae;Paenibacillus;	0	0	0	0	14	0	3
Otu0098	Bacteria;Cyanobacteria;Chloroplast;Chloroplast_or;Chloroplast_fa;Chloroplast_ge;	8	0	9	0	0	0	0
Otu0099	Bacteria;Proteobacteria;Proteobacteria_unclassified;Proteobacteria_unclassified;Proteobacteria_unclassified;	0	0	0	7	10	0	0
Otu0100	Bacteria;Actinobacteria;Actinobacteria;Micromonosporales;Micromonosporaceae;Actinoplanes;	15	0	0	0	0	0	0
Otu0101	Bacteria;Proteobacteria;Alphaproteobacteria;Caulobacterales;Caulobacteraceae;	0	0	0	0	15	0	0
Otu0102	Bacteria;Actinobacteria;Actinobacteria;Frankiales;Geodermatophilaceae;Blastococcus;	0	0	15	0	0	0	0
Otu0103	Bacteria;Proteobacteria;Alphaproteobacteria;Rhodospirillales;Acetobacteraceae;Roseomonas;	0	0	0	0	12	0	2
Otu0104	Bacteria;Actinobacteria;Actinobacteria;Bifidobacteriales;Bifidobacteriaceae;	0	4	0	0	0	8	2
Otu0105	Bacteria;Actinobacteria;Actinobacteria;Micrococcales;Micrococcaceae;	11	0	2	0	0	0	0
Otu0106	Bacteria;Firmicutes;Bacilli;Bacillales;Paenibacillaceae;Paenibacillus;	0	0	0	10	0	0	3
Otu0107	Bacteria;Firmicutes;Bacilli;Bacillales;Bacillaceae;Bacillus;	7	0	6	0	0	0	0
Otu0108	Bacteria;Firmicutes;Clostridia;Clostridiales;Clostridiaceae_1;Clostridium_sensu_stricto_18;	0	0	0	7	0	6	0
Otu0109	Bacteria;Proteobacteria;Alphaproteobacteria;Rhizobiales;Methylobacteriaceae;Methylobacterium;	6	0	7	0	0	0	0
Otu0110	Bacteria;Cyanobacteria;Chloroplast;Chloroplast_or;Chloroplast_fa;Chloroplast_ge;	0	0	12	0	0	0	0
Otu0111	Bacteria;Firmicutes;Bacilli;Bacillales;Bacillales_unclassified;	0	0	0	8	4	0	0
Otu0112	Bacteria;Cyanobacteria;Chloroplast;Chloroplast_or;Chloroplast_fa;Chloroplast_ge;	0	4	7	0	0	0	0
Otu0113	Bacteria;Firmicutes;Bacilli;Bacillales;Paenibacillaceae;	0	0	0	7	4	0	0
Otu0114	Bacteria;Actinobacteria;Actinobacteria;Streptosporangiales;Streptosporangiaceae;Microbispora;	11	0	0	0	0	0	0
Otu0115	Bacteria;Bacteroidetes;Cytophagia;Cytophagales;Cytophagaceae;Dyadobacter;	0	0	0	0	10	0	0
Otu0116	Bacteria;Bacteroidetes;Cytophagia;Cytophagales;Cytophagaceae;Hymenobacter;	0	10	0	0	0	0	0
Otu0117	Bacteria;Cyanobacteria;Chloroplast;Chloroplast_or;Chloroplast_fa;Chloroplast_ge;	4	2	4	0	0	0	0
Otu0118	Bacteria;Actinobacteria;Actinobacteria_unclassified;Actinobacteria_unclassified;Actinobacteria_unclassified;	0	0	10	0	0	0	0
Otu0119	Bacteria;Cyanobacteria;Chloroplast;Chloroplast_or;Chloroplast_fa;Chloroplast_ge;	0	0	9	0	0	0	0
Otu0120	Bacteria;Firmicutes;Bacilli;Bacillales;Bacillaceae;	0	0	0	5	0	0	4
Otu0121	Bacteria;Actinobacteria;Actinobacteria;Micrococcales;Micrococcaceae;	4	3	2	0	0	0	0
Otu0122	Bacteria;Bacteroidetes;Flavobacteriia;Flavobacteriales;Flavobacteriaceae;	0	0	0	9	0	0	0
Otu0123	Bacteria;Bacteroidetes;Flavobacteriia;Flavobacteriales;Flavobacteriaceae;	0	0	0	0	9	0	0
Otu0124	Bacteria;Actinobacteria;Actinobacteria;Pseudonocardiales;Pseudonocardiaceae;Saccharopolyspora;	9	0	0	0	0	0	0
Otu0125	Bacteria;Cyanobacteria;Chloroplast;Chloroplast_or;Chloroplast_fa;Chloroplast_ge;	0	9	0	0	0	0	0
Otu0126	Bacteria;Proteobacteria;Alphaproteobacteria;Rhizobiales;Rhizobiales_unclassified;	0	8	0	0	0	0	0
Otu0127	Bacteria;Firmicutes;Bacilli;Bacillales;Paenibacillaceae;Paenibacillus;	0	0	0	4	4	0	0
Otu0128	Bacteria;Cyanobacteria;Chloroplast;Chloroplast_or;Chloroplast_fa;Chloroplast_ge;	0	0	8	0	0	0	0
Otu0129	Bacteria;Proteobacteria;Gammaproteobacteria;Pseudomonadales;Pseudomonadaceae;	0	0	0	0	8	0	0
Otu0130	Bacteria;Firmicutes;Bacilli;Bacillales;Bacillaceae;Bacillus;	0	0	0	8	0	0	0
Otu0131	Bacteria;Bacteroidetes;Flavobacteriia;Flavobacteriales;Flavobacteriaceae;Flavobacterium;	0	0	0	0	8	0	0
Otu0132	Bacteria;Proteobacteria;Alphaproteobacteria;Sphingomonadales;Sphingomonadales_unclassified;	8	0	0	0	0	0	0
Otu0133	Bacteria;Proteobacteria;Deltaproteobacteria;Myxococcales;Cystobacteraceae;Melittangium;	8	0	0	0	0	0	0
Otu0134	Bacteria;Firmicutes;Bacilli;Bacillales;Paenibacillaceae;Paenibacillus;	0	0	0	7	0	0	0
Otu0135	Bacteria;Cyanobacteria;Chloroplast;Chloroplast_or;Chloroplast_fa;Chloroplast_ge;	0	0	7	0	0	0	0
Otu0136	Bacteria;Firmicutes;Clostridia;Clostridiales;Peptostreptococcaceae;	4	3	0	0	0	0	0
Otu0137	Bacteria;Cyanobacteria;Chloroplast;Chloroplast_or;Chloroplast_fa;Chloroplast_ge;	0	7	0	0	0	0	0
Otu0138	Bacteria;Proteobacteria;Betaproteobacteria;Burkholderiales;Comamonadaceae;	0	0	0	0	7	0	0
Otu0139	Bacteria;Proteobacteria;Alphaproteobacteria;Rickettsiales;Mitochondria;Mitochondria_ge;	4	0	3	0	0	0	0
Otu0140	Bacteria;Bacteroidetes;Cytophagia;Cytophagales;Cytophagaceae;Hymenobacter;	7	0	0	0	0	0	0
Otu0141	Bacteria;Proteobacteria;Alphaproteobacteria;Rhodospirillales;Acetobacteraceae;Asaia;	0	6	0	0	0	0	0
Otu0142	Bacteria;Firmicutes;Bacilli;Bacillales;Staphylococcaceae;Staphylococcus;	6	0	0	0	0	0	0
Otu0143	Bacteria;Firmicutes;Bacilli;Lactobacillales;Lactobacillales_unclassified;	6	0	0	0	0	0	0
Otu0144	Bacteria;Proteobacteria;Alphaproteobacteria;Rhodospirillales;Rhodospirillaceae;Skermanella;	0	6	0	0	0	0	0
Otu0145	Bacteria;Actinobacteria;Thermoleophilia;Solirubrobacterales;FFCH13075;FFCH13075_ge;	0	6	0	0	0	0	0
Otu0146	Bacteria;Actinobacteria;Actinobacteria;Bifidobacteriales;Bifidobacteriaceae;	0	2	0	0	0	4	0
Otu0147	Bacteria;Actinobacteria;Actinobacteria;Corynebacteriales;Dietziaceae;Dietzia;	0	0	6	0	0	0	0
Otu0148	Bacteria;Cyanobacteria;Chloroplast;Chloroplast_or;Chloroplast_fa;Chloroplast_ge;	4	2	0	0	0	0	0
Otu0149	Bacteria;Cyanobacteria;Chloroplast;Chloroplast_or;Chloroplast_fa;Chloroplast_ge;	0	6	0	0	0	0	0

Otu0150	Bacteria;Proteobacteria;Alphaproteobacteria;Sphingomonadales;Sphingomonadaceae;Sphingomonas;	2	4	0	0	0	0	0
Otu0151	Bacteria;Actinobacteria;Actinobacteria;Propionibacteriales;Nocardioidaceae;Nocardioides;	0	6	0	0	0	0	0
Otu0152	Bacteria;Firmicutes;Bacilli;Bacillales;Bacillaceae;Bacillus;	6	0	0	0	0	0	0
Otu0153	Bacteria;Actinobacteria;Actinobacteria;Streptosporangiales;Nocardiopsaceae;Nocardiopsis;	6	0	0	0	0	0	0
Otu0154	Bacteria;Chloroflexi;Thermomicrobia;JG30-KF-CM45;JG30-KF-CM45_fa;JG30-KF-CM45_ge;	0	0	5	0	0	0	0
Otu0155	Bacteria;Cyanobacteria;Chloroplast;Chloroplast_or;Chloroplast_fa;Chloroplast_ge;	0	0	5	0	0	0	0
Otu0156	Bacteria;Firmicutes;Bacilli;Bacillales;Planococcaceae;Planomicrobium;	5	0	0	0	0	0	0
Otu0157	Bacteria;Firmicutes;Bacilli;Bacillales;Bacillaceae;Bacillus;	5	0	0	0	0	0	0
Otu0158	Bacteria;Cyanobacteria;Chloroplast;Chloroplast_or;Chloroplast_fa;Chloroplast_ge;	0	0	5	0	0	0	0
Otu0159	Bacteria;Actinobacteria;Actinobacteria;Frankiales;Geodermatophilaceae;Geodermatophilus;	0	0	5	0	0	0	0
Otu0160	Bacteria;Firmicutes;Bacilli;Bacillales;Bacillaceae;Oceanobacillus;	0	0	0	5	0	0	0
Otu0161	Bacteria;Cyanobacteria;Cyanobacteria;SubsectionIII;FamilyI;Phormidium;	0	5	0	0	0	0	0
Otu0162	Bacteria;Proteobacteria;Alphaproteobacteria;Rhodobacterales;Rhodobacteraceae;Rubellimicrobium;	0	0	5	0	0	0	0
Otu0163	Bacteria;Proteobacteria;Alphaproteobacteria;Rhodospirillales;Rhodospirillales_Incertae_Sedis;Geminicoccus;	5	0	0	0	0	0	0
Otu0164	Bacteria;Proteobacteria;Gammaproteobacteria;Pseudomonadales;Pseudomonadaceae;Pseudomonadaceae_ge;	5	0	0	0	0	0	0
Otu0165	Bacteria;Proteobacteria;Alphaproteobacteria;Rhodospirillales;Acetobacteraceae;	0	0	5	0	0	0	0
Otu0166	Bacteria;Proteobacteria;Alphaproteobacteria;Rhodospirillales;Acetobacteraceae;	5	0	0	0	0	0	0
Otu0167	Bacteria;Cyanobacteria;Chloroplast;Chloroplast_or;Chloroplast_fa;Chloroplast_ge;	3	2	0	0	0	0	0
Otu0168	Bacteria;Cyanobacteria;Cyanobacteria;SubsectionIII;FamilyI;	0	5	0	0	0	0	0
Otu0169	Bacteria;Cyanobacteria;Chloroplast;Chloroplast_or;Chloroplast_fa;Chloroplast_ge;	0	0	5	0	0	0	0
Otu0170	Bacteria;Actinobacteria;Actinobacteria;Micrococcales;Microbacteriaceae;Agromyces;	2	0	0	0	3	0	0
Otu0171	Bacteria;Cyanobacteria;Chloroplast;Chloroplast_or;Chloroplast_fa;Chloroplast_ge;	0	0	5	0	0	0	0
Otu0172	Bacteria;Firmicutes;Bacilli;Lactobacillales;Lactobacillaceae;Lactobacillus;	2	3	0	0	0	0	0
Otu0173	Bacteria;Proteobacteria;Alphaproteobacteria;Rhodospirillales;Rhodospirillaceae;Azospirillum;	0	0	0	0	5	0	0
Otu0174	Bacteria;Actinobacteria;Actinobacteria;Micrococcales;Micrococcaceae;	5	0	0	0	0	0	0
Otu0175	Bacteria;Cyanobacteria;Chloroplast;Chloroplast_or;Chloroplast_fa;Chloroplast_ge;	0	0	5	0	0	0	0
Otu0176	Bacteria;Cyanobacteria;Chloroplast;Chloroplast_or;Chloroplast_fa;Chloroplast_ge;	0	0	5	0	0	0	0
Otu0177	Bacteria;Proteobacteria;Alphaproteobacteria;Rhizobiales;Xanthobacteraceae;	0	5	0	0	0	0	0
Otu0178	Bacteria;Cyanobacteria;Chloroplast;Chloroplast_or;Chloroplast_fa;Chloroplast_ge;	5	0	0	0	0	0	0
Otu0179	Bacteria;Bacteroidetes;Flavobacteriia;Flavobacteriales;Flavobacteriaceae;Flavobacterium;	0	0	0	0	5	0	0
Otu0180	Bacteria;Bacteroidetes;Sphingobacteriia;Sphingobacteriales;Sphingobacteriaceae;Mucilaginibacter;	0	0	0	0	5	0	0
Otu0181	Bacteria;Proteobacteria;Betaproteobacteria;Burkholderiales;Comamonadaceae;	0	5	0	0	0	0	0
Otu0182	Bacteria;Cyanobacteria;Chloroplast;Chloroplast_or;Chloroplast_fa;Chloroplast_ge;	0	0	4	0	0	0	0
Otu0183	Bacteria;Firmicutes;Bacilli;Bacillales;Bacillaceae;	4	0	0	0	0	0	0
Otu0184	Bacteria;Firmicutes;Bacilli;Bacillales;Paenibacillaceae;Paenibacillus;	0	0	0	0	0	0	4
Otu0185	Bacteria;Actinobacteria;Actinobacteria;Micrococcales;Microbacteriaceae;Rathayibacter;	0	0	4	0	0	0	0
Otu0186	Bacteria;Proteobacteria;Betaproteobacteria;Burkholderiales;Comamonadaceae;	0	0	0	0	0	0	4
Otu0187	Bacteria;Cyanobacteria;Chloroplast;Chloroplast_or;Chloroplast_fa;Chloroplast_ge;	0	0	4	0	0	0	0
Otu0188	Bacteria;Actinobacteria;Actinobacteria;Micromonosporales;Micromonosporaceae;	4	0	0	0	0	0	0
Otu0189	Bacteria;Cyanobacteria;Chloroplast;Chloroplast_or;Chloroplast_fa;Chloroplast_ge;	0	4	0	0	0	0	0
Otu0190	Bacteria;Actinobacteria;Actinobacteria;Frankiales;Geodermatophilaceae;Geodermatophilus;	0	4	0	0	0	0	0
Otu0191	Bacteria;Proteobacteria;Alphaproteobacteria;Rhizobiales;Rhizobiaceae;	0	4	0	0	0	0	0
Otu0192	Bacteria;Proteobacteria;Betaproteobacteria;Burkholderiales;Comamonadaceae;	0	0	0	4	0	0	0
Otu0193	Bacteria;Proteobacteria;Gammaproteobacteria;Pseudomonadales;Pseudomonadaceae;	0	0	0	0	0	2	2
Otu0194	Bacteria;Proteobacteria;Gammaproteobacteria;Pseudomonadales;Pseudomonadaceae;	0	0	0	2	0	0	2
Otu0195	Bacteria;Proteobacteria;Gammaproteobacteria;Xanthomonadales;Xanthomonadales_Incertae_Sedis;Steroidobacter;	0	0	4	0	0	0	0
Otu0196	Bacteria;Actinobacteria;Actinobacteria;Actinobacteria_unclassified;Actinobacteria_unclassified;	4	0	0	0	0	0	0
Otu0197	Bacteria;Cyanobacteria;Chloroplast;Chloroplast_or;Chloroplast_fa;Chloroplast_ge;	4	0	0	0	0	0	0
Otu0198	Bacteria;Proteobacteria;Alphaproteobacteria;Rhizobiales;Methylobacteriaceae;Microvirga;	2	2	0	0	0	0	0
Otu0199	Bacteria;Proteobacteria;Proteobacteria_unclassified;Proteobacteria_unclassified;Proteobacteria_unclassified;	0	0	0	0	4	0	0
Otu0200	Bacteria;Firmicutes;Clostridia;Clostridiales;Peptostreptococcaceae;	0	0	4	0	0	0	0
Otu0201	Bacteria;Bacteroidetes;Flavobacteriia;Flavobacteriales;Flavobacteriaceae;	0	0	0	0	4	0	0
Otu0202	Bacteria;Firmicutes;Bacilli;Lactobacillales;Lactobacillales_unclassified;	4	0	0	0	0	0	0
Otu0203	Bacteria;Proteobacteria;Gammaproteobacteria;Xanthomonadales;Xanthomonadales_Incertae_Sedis;Acidibacter;	0	0	3	0	0	0	0
Otu0204	Bacteria;Bacteroidetes;Sphingobacteriia;Sphingobacteriales;Chitinophagaceae;Flavisolibacter;	3	0	0	0	0	0	0
Otu0205	Bacteria;Acidobacteria;Acidobacteria;Subgroup_3;Unknown_Family;Bryobacter;	3	0	0	0	0	0	0
Otu0206	Bacteria;Chloroflexi;Thermomicrobia;JG30-KF-CM45;JG30-KF-CM45_fa;JG30-KF-CM45_ge;	0	3	0	0	0	0	0
Otu0207	Bacteria;Proteobacteria;Proteobacteria_unclassified;Proteobacteria_unclassified;Proteobacteria_unclassified;	0	0	0	0	3	0	0
Otu0208	Bacteria;Bacteroidetes;Flavobacteriia;Flavobacteriales;Flavobacteriaceae;Epilithonimonas;	0	0	0	0	0	0	3
Otu0209	Bacteria;Bacteroidetes;Flavobacteriia;Flavobacteriales;Flavobacteriaceae;Flavobacterium;	0	0	0	0	3	0	0
Otu0210	Bacteria;Cyanobacteria;Chloroplast;Chloroplast_or;Chloroplast_fa;Chloroplast_ge;	3	0	0	0	0	0	0
Otu0211	Bacteria;Cyanobacteria;Chloroplast;Chloroplast_or;Chloroplast_fa;Chloroplast_ge;	0	0	3	0	0	0	0
Otu0212	Bacteria;Proteobacteria;Gammaproteobacteria;Pseudomonadales;Moraxellaceae;Acinetobacter;	3	0	0	0	0	0	0
Otu0213	Bacteria;Firmicutes;Bacilli;Bacillales;Planococcaceae;Solibacillus;	0	3	0	0	0	0	0
Otu0214	Bacteria;Cyanobacteria;Cyanobacteria_unclassified;Cyanobacteria_unclassified;Cyanobacteria_unclassified;	0	3	0	0	0	0	0
Otu0215	Bacteria;Cyanobacteria;Chloroplast;Chloroplast_or;Chloroplast_fa;Chloroplast_ge;	0	3	0	0	0	0	0
Otu0216	Bacteria;Proteobacteria;Alphaproteobacteria;Rickettsiales;Rickettsiales_Incertae_Sedis;Candidatus_Captivus;	0	0	0	0	3	0	0
Otu0217	Bacteria;Firmicutes;Bacilli;Bacillales;Bacillaceae;Alkalibacillus;	3	0	0	0	0	0	0
Otu0218	Bacteria;Actinobacteria;Actinobacteria;Micrococcales;Brevibacteriaceae;Brevibacterium;	0	3	0	0	0	0	0
Otu0219	Bacteria;Firmicutes;Erysipelotrichia;Erysipelotrichales;Erysipelotrichaceae;uncultured;	0	0	0	0	3	0	0
Otu0220	Bacteria;Proteobacteria;Betaproteobacteria;Burkholderiales;Comamonadaceae;	0	0	0	0	3	0	0
Otu0221	Bacteria;Cyanobacteria;Chloroplast;Chloroplast_or;Chloroplast_fa;Chloroplast_ge;	3	0	0	0	0	0	0
Otu0222	Bacteria;Proteobacteria;Alphaproteobacteria;Rhizobiales;Methylobacteriaceae;Methylobacterium;	3	0	0	0	0	0	0
Otu0223	Bacteria;Cyanobacteria;Chloroplast;Chloroplast_or;Chloroplast_fa;Chloroplast_ge;	0	3	0	0	0	0	0
Otu0224	Bacteria;Cyanobacteria;Chloroplast;Chloroplast_or;Chloroplast_fa;Chloroplast_ge;	0	0	3	0	0	0	0
Otu0225	Bacteria;Proteobacteria;Alphaproteobacteria;Rhizobiales;Rhizobiaceae;Kaistia;	0	0	0	0	3	0	0

Otu0226	Bacteria;Proteobacteria;Alphaproteobacteria;Rhizobiales;Bradyrhizobiaceae;Bosea;	0	3	0	0	0	0	0
Otu0227	Bacteria;Firmicutes;Clostridia;Clostridiales;Clostridiaceae_1;Clostridium_sensu_stricto_13;	3	0	0	0	0	0	0
Otu0228	Bacteria;Proteobacteria;Betaproteobacteria;Burkholderiales;Comamonadaceae;	0	0	0	0	0	0	3
Otu0229	Bacteria;Actinobacteria;Actinobacteria;Micrococcales;Microbacteriaceae;Agromyces;	0	0	0	0	3	0	0
Otu0230	Bacteria;Proteobacteria;Alphaproteobacteria;Rickettsiales;Anaplasmataceae;Wolbachia;	3	0	0	0	0	0	0
Otu0231	Bacteria;Proteobacteria;Alphaproteobacteria;Sphingomonadales;Sphingomonadaceae;	0	3	0	0	0	0	0
Otu0232	Bacteria;Actinobacteria;Actinobacteria;Propionibacteriales;Propionibacteriaceae;Microlunatus;	0	0	3	0	0	0	0
Otu0233	Bacteria;Proteobacteria;Gammaproteobacteria;Oceanospirillales;Halomonadaceae;Carnimonas;	3	0	0	0	0	0	0
Otu0234	Bacteria;Cyanobacteria;Chloroplast;Chloroplast_or;Chloroplast_fa;Chloroplast_ge;	3	0	0	0	0	0	0
Otu0235	Bacteria;Cyanobacteria;Chloroplast;Chloroplast_or;Chloroplast_fa;Chloroplast_ge;	3	0	0	0	0	0	0
Otu0236	Bacteria;Firmicutes;Bacilli;Bacillales;Paenibacillaceae;Paenibacillus;	0	0	0	0	0	0	3
Otu0237	Bacteria;Firmicutes;Bacilli;Bacillales;Paenibacillaceae;Paenibacillus;	0	0	0	0	0	0	3
Otu0238	Bacteria;Proteobacteria;Alphaproteobacteria;Rhizobiales;Rhizobiales_unclassified;	0	3	0	0	0	0	0
Otu0239	Bacteria;Proteobacteria;Gammaproteobacteria;Oceanospirillales;Halomonadaceae;Zymobacter;	0	3	0	0	0	0	0
Otu0240	Bacteria;Firmicutes;Bacilli;Bacillales;Bacillaceae;Bacillus;	0	0	3	0	0	0	0
Otu0241	Bacteria;Proteobacteria;Gammaproteobacteria;Pseudomonadales;Pseudomonadaceae;	0	0	0	0	3	0	0
Otu0242	Bacteria;Firmicutes;Bacilli;Lactobacillales;Enterococcaceae;	0	0	0	0	0	0	2
Otu0243	Bacteria;Actinobacteria;Actinobacteria;Kineosporiales;Kineosporiaceae;Kineosporia;	0	2	0	0	0	0	0
Otu0244	Bacteria;Actinobacteria;Thermoleophilia;Solirubrobacterales;Solirubrobacteraceae;Solirubrobacter;	0	2	0	0	0	0	0
Otu0245	Bacteria;Actinobacteria;Actinobacteria;Micrococcales;Microbacteriaceae;	0	0	0	0	0	0	2
Otu0246	Bacteria;Proteobacteria;Gammaproteobacteria;Orbales;Orbaceae;Candidatus_Schmidhempelia;	0	0	2	0	0	0	0
Otu0247	Bacteria;Proteobacteria;Betaproteobacteria;Burkholderiales;Comamonadaceae;	0	0	0	0	2	0	0
Otu0248	Bacteria;Firmicutes;Bacilli;Lactobacillales;Leuconostocaceae;Weissella;	0	2	0	0	0	0	0
Otu0249	Bacteria;Bacteroidetes;Cytophagia;Cytophagales;Cytophagaceae;Dyadobacter;	0	0	0	0	2	0	0
Otu0250	Bacteria;Actinobacteria;Thermoleophilia;Solirubrobacterales;Solirubrobacteraceae;Solirubrobacter;	0	2	0	0	0	0	0
Otu0251	Bacteria;Actinobacteria;Actinobacteria;Propionibacteriales;Nocardioidaceae;Nocardioides;	0	2	0	0	0	0	0
Otu0252	Bacteria;Planctomycetes;Planctomycetacia;Planctomycetales;Planctomycetaceae;	2	0	0	0	0	0	0
Otu0253	Bacteria;Bacteroidetes;Cytophagia;Cytophagales;Cytophagaceae;Hymenobacter;	0	2	0	0	0	0	0
Otu0254	Bacteria;Bacteroidetes;Sphingobacteriia;Sphingobacteriales;Sphingobacteriaceae;Pedobacter;	2	0	0	0	0	0	0
Otu0255	Bacteria;Firmicutes;Bacilli;Bacillales;Paenibacillaceae;Paenibacillus;	0	0	0	2	0	0	0
Otu0256	Bacteria;Firmicutes;Bacilli;Bacillales;Family_XII;Exiguobacterium;	0	0	2	0	0	0	0
Otu0257	Bacteria;Firmicutes;Bacilli;Lactobacillales;Lactobacillales_unclassified;	0	0	0	2	0	0	0
Otu0258	Bacteria;Proteobacteria;Deltaproteobacteria;Myxococcales;Sandaracinaceae;	2	0	0	0	0	0	0
Otu0259	Bacteria;Firmicutes;Bacilli;Bacillales;Planococcaceae;	0	0	0	0	2	0	0
Otu0260	Bacteria;Firmicutes;Bacilli;Lactobacillales;Enterococcaceae;Enterococcus;	0	0	0	0	0	2	0
Otu0261	Bacteria;Proteobacteria;Alphaproteobacteria;Rhizobiales;Rhizobiaceae;Rhizobium;	0	0	0	0	2	0	0
Otu0262	Bacteria;Proteobacteria;Alphaproteobacteria;Caulobacterales;Caulobacteraceae;Brevundimonas;	0	0	0	0	0	0	2
Otu0263	Bacteria;Firmicutes;Bacilli;Lactobacillales;Lactobacillaceae;Lactobacillus;	0	2	0	0	0	0	0
Otu0264	Bacteria;Cyanobacteria;Chloroplast;Chloroplast_or;Chloroplast_fa;Chloroplast_ge;	2	0	0	0	0	0	0
Otu0265	Bacteria;Actinobacteria;Actinobacteria;Streptosporangiales;Thermomonosporaceae;Actinomadura;	2	0	0	0	0	0	0
Otu0266	Bacteria;Proteobacteria;Gammaproteobacteria;Pseudomonadales;Pseudomonadaceae;	0	0	0	0	0	2	0
Otu0267	Bacteria;Proteobacteria;Alphaproteobacteria;Sphingomonadales;Sphingomonadales_unclassified;	0	2	0	0	0	0	0
Otu0268	Bacteria;Bacteroidetes;Sphingobacteriia;Sphingobacteriales;Chitinophagaceae;Flavisolibacter;	0	2	0	0	0	0	0
Otu0269	Bacteria;Actinobacteria;Actinobacteria;Corynebacteriales;Mycobacteriaceae;Mycobacterium;	2	0	0	0	0	0	0
Otu0270	Bacteria;Cyanobacteria;Chloroplast;Chloroplast_or;Chloroplast_fa;Chloroplast_ge;	2	0	0	0	0	0	0
Otu0271	Bacteria;Cyanobacteria;Chloroplast;Chloroplast_or;Chloroplast_fa;Chloroplast_ge;	0	0	2	0	0	0	0
Otu0272	Bacteria;Gemmatimonadetes;Gemmatimonadetes;Gemmatimonadales;Gemmatimonadaceae;	2	0	0	0	0	0	0
Otu0273	Bacteria;Deinococcus-Thermus;Deinococci;Deinococcales;Deinococcaceae;Deinococcus;	0	0	2	0	0	0	0
Otu0274	Bacteria;Actinobacteria;Actinobacteria;Kineosporiales;Kineosporiaceae;	0	2	0	0	0	0	0
Otu0275	Bacteria;Proteobacteria;Betaproteobacteria;Burkholderiales;Comamonadaceae;Delftia;	0	0	0	0	2	0	0
Otu0276	Bacteria;Firmicutes;Bacilli;Lactobacillales;Lactobacillales_unclassified;	0	0	0	0	0	2	0
Otu0277	Bacteria;Proteobacteria;Betaproteobacteria;Burkholderiales;Comamonadaceae;	0	0	0	2	0	0	0
Otu0278	Bacteria;Proteobacteria;Gammaproteobacteria;Oceanospirillales;Halomonadaceae;Halomonas;	2	0	0	0	0	0	0
Otu0279	Bacteria;Actinobacteria;Actinobacteria;Propionibacteriales;Nocardioidaceae;	2	0	0	0	0	0	0
Otu0280	Bacteria;Firmicutes;Bacilli;Bacillales;Bacillaceae;Bacillus;	2	0	0	0	0	0	0
Otu0281	Bacteria;Bacteroidetes;Flavobacteriia;Flavobacteriales;Flavobacteriaceae;	0	0	0	2	0	0	0
Otu0282	Bacteria;Proteobacteria;Betaproteobacteria;Burkholderiales;Comamonadaceae;Xenophilus;	0	0	0	0	2	0	0
Otu0283	Bacteria;Actinobacteria;Actinobacteria;Actinobacteria_unclassified;Actinobacteria_unclassified;	2	0	0	0	0	0	0
Otu0284	Bacteria;Actinobacteria;Actinobacteria;Streptosporangiales;Nocardiopsaceae;Nocardiopsis;	2	0	0	0	0	0	0
Otu0285	Bacteria;Actinobacteria;Actinobacteria;Micrococcales;Microbacteriaceae;	0	0	0	0	2	0	0
Otu0286	Bacteria;Firmicutes;Bacilli;Bacillales;Planococcaceae;Planomicrobium;	2	0	0	0	0	0	0
Otu0287	Bacteria;Proteobacteria;Alphaproteobacteria;Rhodospirillales;Acetobacteraceae;Roseomonas;	0	0	0	0	0	0	2
Otu0288	Bacteria;Cyanobacteria;Chloroplast;Chloroplast_or;Chloroplast_fa;Chloroplast_ge;	2	0	0	0	0	0	0
Otu0289	Bacteria;Proteobacteria;Betaproteobacteria;Betaproteobacteria_unclassified;Betaproteobacteria_unclassified;	0	0	0	0	0	0	2
Otu0290	Bacteria;Bacteroidetes;Sphingobacteriia;Sphingobacteriales;Sphingobacteriaceae;Sphingobacterium;	0	0	0	0	0	0	2
Otu0291	Bacteria;Saccharibacteria;Saccharibacteria_cl;Saccharibacteria_or;Saccharibacteria_fa;Saccharibacteria_ge;	0	2	0	0	0	0	0
Otu0292	Bacteria;Cyanobacteria;Chloroplast;Chloroplast_or;Chloroplast_fa;Chloroplast_ge;	2	0	0	0	0	0	0
Otu0293	Bacteria;Cyanobacteria;Chloroplast;Chloroplast_or;Chloroplast_fa;Chloroplast_ge;	2	0	0	0	0	0	0
Otu0294	Bacteria;Firmicutes;Bacilli;Lactobacillales;Lactobacillales_unclassified;	0	0	0	0	2	0	0
Otu0295	Bacteria;Actinobacteria;Actinobacteria;Corynebacteriales;Corynebacteriaceae;Corynebacterium_1;	2	0	0	0	0	0	0
Otu0296	Bacteria;Actinobacteria;Actinobacteria;Micromonosporales;Micromonosporaceae;	0	0	0	0	0	2	0
Otu0297	Bacteria;Proteobacteria;Alphaproteobacteria;Rhodospirillales;Acetobacteraceae;Roseomonas;	0	2	0	0	0	0	0
Otu0298	Bacteria;Cyanobacteria;Chloroplast;Chloroplast_or;Chloroplast_fa;Chloroplast_ge;	0	0	2	0	0	0	0
Otu0299	Bacteria;Firmicutes;Bacilli;Lactobacillales;Carnobacteriaceae;Carnobacterium;	2	0	0	0	0	0	0
Otu0300	Bacteria;Proteobacteria;Gammaproteobacteria;Oceanospirillales;Halomonadaceae;Carnimonas;	0	2	0	0	0	0	0
Otu0301	Bacteria;Firmicutes;Erysipelotrichia;Erysipelotrichales;Erysipelotrichaceae;Turicibacter;	0	0	0	0	0	0	2

Otu0302	Bacteria;Verrucomicrobia;Verrucomicrobiae;Verrucomicrobiales;Verrucomicrobiaceae;Haloferula;	0	0	0	0	2	0	0
Otu0303	Bacteria;Bacteroidetes;Flavobacteriia;Flavobacteriales;Flavobacteriaceae;	0	0	0	2	0	0	0
Otu0304	Bacteria;Actinobacteria;Thermoleophilia;Solirubrobacterales;480-2;480-2_ge;	2	0	0	0	0	0	0
Otu0305	Bacteria;Proteobacteria;Alphaproteobacteria;Rhizobiales;Rhizobiales_unclassified;	2	0	0	0	0	0	0
Otu0306	Bacteria;Proteobacteria;Alphaproteobacteria;Rhizobiales;Bradyrhizobiaceae;Bosea;	2	0	0	0	0	0	0
Otu0307	Bacteria;Proteobacteria;Gammaproteobacteria;Xanthomonadales;Xanthomonadaceae;Stenotrophomonas;	0	0	0	0	2	0	0
Otu0308	Bacteria;Actinobacteria;Actinobacteria_unclassified;Actinobacteria_unclassified;Actinobacteria_unclassified;	0	2	0	0	0	0	0
Otu0309	Bacteria;Actinobacteria;Actinobacteria;Micrococcales;Dermabacteraceae;Brachybacterium;	0	0	2	0	0	0	0
Otu0310	Bacteria;Firmicutes;Bacilli;Lactobacillales;Lactobacillales_unclassified;	0	0	0	0	0	2	0
Otu0311	Bacteria;Proteobacteria;Betaproteobacteria;Betaproteobacteria_unclassified;Betaproteobacteria_unclassified;	0	0	0	0	0	0	2
Otu0312	Bacteria;Cyanobacteria;Chloroplast;Chloroplast_or;Chloroplast_fa;Chloroplast_ge;	0	2	0	0	0	0	0

Table S2. Shannon's diversity (H') and Pielou's evenness (J') of bacterial and fungal community isolated from Source K (N = 3) and Source B (N = 4)

	K1	K2	K3	Mean	SE		B1	B2	B3	B4	Mean	SE	*P*
Bacterial H'	1.49	0.49	0.75	0.91	0.3		2.53	2.38	2.64	2.58	2.53	0.06	0.01
Bacterial J'	0.32	0.11	0.18	0.2	0.06		0.58	0.5	0.62	0.59	0.57	0.02	< 0.01
Fungal H'	1.55	1.24	0.93	1.24	0.18		0.85	0.88	0.69	0.64	0.77	0.06	0.04
Fungal J'	0.44	0.34	0.3	0.36	0.04		0.28	0.27	0.24	0.19	0.24	0.02	0.04

Table S3. Distribution of sequencing reads of bacterial and fungal taxa at the family and order rank respectively. Source K N = 3; Source B N = 4.

		Source K		Source B	
	Family	Mean	SE	Mean	SE
Bacteria community	Planococcaceae	0.04%	0.03	4.01%	1.03
	Lactobacillaceae	77.11%	19.16	10.79%	4.42
	Enterococcaceae	0.00%	0.00	2.18%	1.07
	Acetobacteraceae	0.03%	0.01	1.94%	1.38
	Brucellaceae	0.00%	0.00	3.31%	1.08
	Comamonadaceae	0.05%	0.01	33.97%	3.39
	Xanthomonadaceae	0.04%	0.04	9.41%	1.93
	Pseudomonadaceae	0.66%	0.59	10.28%	2.47
	Flavobacteriaceae	0.01%	0.01	8.59%	3.34
	Sphingobacteriaceae	0.01%	0.00	9.28%	2.69
	Streptomycetaceae	16.25%	16.24	0.01%	0.01
	Bifidobacteriaceae	4.72%	1.83	1.14%	0.55
	Other bacteria	1.08%	0.51	5.08%	0.89
		Source K		Source B	
	Order	Mean	SE	Mean	SE
Fungal community	Saccharomycetales	56.14%	19.74	99.80%	0.09
	Ascosphaerales	35.64%	21.67	0.11%	0.08
	Eurotiales	1.42%	1.42	0.09%	0.04
	Sordariales	2.95%	2.95	0.00%	0.00
	Pleosporales	2.57%	1.45	0.00%	0.00
	Other fungi	1.27%	0.44	0.00%	0.00

Table S4. Colony demography and mother queen weights of hives from Source K (N = 3) and Source B (N = 4)

	Source K		Source B	
	Mean	SE	Mean	SE
Initial number of workers	28.00	3.06	51.50	8.61
Final number of workers	67.67	4.26	180.25	28.51
Percent change in number of workers	147.51	29.50	255.08	18.56
Initial weight of mother queen (g)	0.77	0.05	0.80	0.09
Final weight of mother queen (g)	0.76	0.02	0.81	0.07
Percent change in weight of mother queen (g)	-1.45	5.68	2.44	3.48

Table S5. Top blast hit (Mothur) of genus *Ascosphaera* isolated in this study

Description	%ID	Accession	E- value
Ascosphaera apis	96%	GQ867785.1	5e-166
Ascosphaera pollenicola	96%	GQ867791.1	9e-163
Ascosphaera major	96%	GQ867789.1	9e-163
Ascosphaera larvis	96%	JX268535.1	9e-163
Ascosphaera variegata	96%	JX268538.1	3e-162
Ascosphaera aggregata	94%	GQ867784.1	3e-162
Ascosphaera flava	96%	GQ867788.1	1e-161
Ascosphaera proliperda	95%	GQ867792.1	4e-162
Ascosphaera osmophila	94%	GQ867790.1	5e-160
